# Transport Properties of Doped Wide Band Gap Layered Oxychalcogenide Semiconductors Sr_2_GaO_3_Cu*Ch*, Sr_2_ScO_3_Cu*Ch*, and Sr_2_InO_3_Cu*Ch* (*Ch* = S or Se)

**DOI:** 10.1021/acs.chemmater.4c02760

**Published:** 2024-11-14

**Authors:** Zahida Malik, Liam Kemp, Bastien F. Grosso, Daniel W. Davies, David O. Scanlon, Geoffrey Hyett

**Affiliations:** † School of Chemistry and Chemical Engineering, Faculty of Engineering and Physical Sciences, University of Southampton, Highfield Campus, Southampton SO17 1BJ, U.K.; ‡ School of Chemistry, University of Birmingham, Edgbaston, Birmingham B15 2TT, U.K.; § Department of Chemistry, University College London, London WC1H 0AJ, U.K.

## Abstract

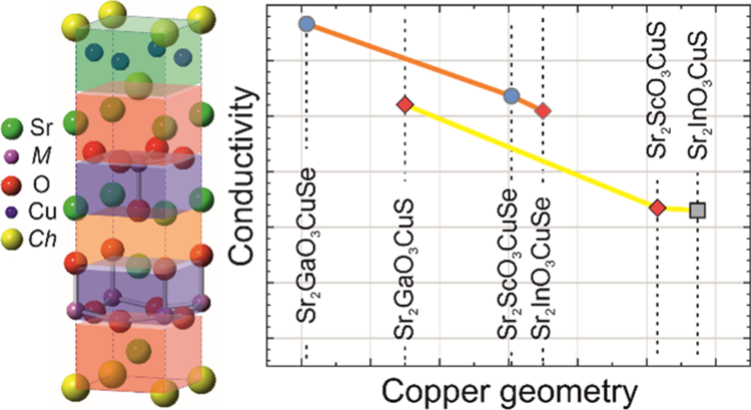

The structural, electrical, and optical properties of a series of six layered oxychalcogenides with the general formula Sr_2_*M*O_3_Cu*Ch*, where M = Ga, Sc, or In and *Ch* = S or Se, have been investigated. From this set, we report the structure and properties of Sr_2_GaO_3_CuSe for the first time, as well as the full structural details of Sr_2_ScO_3_CuSe, which have not previously been available. A systematic study of the suitability of all of the Sr_2_*M*O_3_Cu*Ch* phases as *p*-type conductors has been carried out, after doping with both sodium and potassium to a nominal composition of *A*_0.05_Sr_1.95_*M*O_3_Cu*Ch*, (*A* = Na or K), to increase the hole carrier concentration. Density functional theory calculations were used to determine the electronic band structure and predict the transport properties, while optical properties were determined using UV–vis spectroscopy, and structures were confirmed using Rietveld refinement against powder X-ray diffraction data. Room-temperature conductivity measurements were carried out on both pristine samples and doped samples, 18 compositions in total, using four-point probe measurements. We found that the most conductive sample was K_0.05_Sr_1.95_GaO_3_CuSe, with a measured conductivity of 0.46 S cm^–1^, collected from a sintered pellet. We have also been able to identify a relationship between the conductivity and the geometry of the copper chalcogenide layer within the Sr_2_*M*O_3_Cu*Ch* series of compounds. As this geometry can be controlled through the material composition, the identification of this structure–property relationship highlights a route to the selection and identification of materials with even higher conductivities.

## Introduction

Transparent conducting oxides (TCOs) are key materials for modern technology.^1−5^ They combine the transparency of a wide band gap semiconductor with good electrical conductivity, nearing that of a metal. This is achieved through degenerate doping of semiconductor materials with highly mobile charge carriers, i.e., doping to such an extent that the Fermi level shifts into the band of the material giving metallic-like conductivity, typically requiring ∼10^21^ cm^–3^ carriers.^6^ However, all of the commercially viable materials, (AZO, ITO, and FTO) are *n*-type conductors, leaving a gap in the materials space for an effective *p*-type transparent conductor.^7−10^ The most productive area of research to identify a wide band gap *p*-type conductor has been that of mixed-anion layered copper chalcogenides such as LaOCu*Ch* (*Ch* = S or Se), Sr_3_Sc_2_O_5_Cu_2_S_2_, Sr_2_ZnO_2_Cu_2_S_2_, and Sr_2_GaO_3_CuS.^11−17^ After doping with alkali metals to introduce holes these have reported conductivities ranging from 2.4 × 10^–2^ to 9.1 × 10^2^ S cm^–1^, but with no single material identified combining sufficient conductivity *and* transparency to rival the top *n*-type transparent conductor materials.

The shared structural feature responsible for the *p*-type conductivity in all of these oxychalcogenides is the *anti-litharge* copper chalcogenide layer composed of edge-sharing Cu*Ch*_4_ tetrahedra separated into alternating stripes by metal oxide layers. The most simple example is LaOCuS, where the tetrahedral chalcogenide [CuS]^−^ layers are separated by [LaO]^+^ oxide layers. In all of the layered oxychalcogenides, the overlap of the Cu 3d orbitals and the chalcogenide 3p or 4p orbitals leads to a dispersive band with high hole mobility at the valence band maximum (VBM). Intrinsic copper vacancies can introduce holes into the VBM, or they can be intentionally introduced by aliovalent doping of cation sites within the oxide layer.^12^ The conductivity therefore occurs primarily through the *anti-litharge* copper chalcogenide layer which, as indicated above, is found in a wide range of layered mixed-anion materials, with the most common having compositions of *A*_2_*M*O_2_Cu_2_*Ch*_2_,^18,19^*A*_3_*M*_2_O_5_Cu_2_*Ch*_2_,^20,21^ or *A*_2_*M*O_3_Cu*Ch*,^22,23^ where *A* is an alkaline earth metal and *M* is a transition or p block metal. An excellent summary of these structure types and the relationships between them can be found in the review by Clarke et al.^24^ We have published a number of papers investigating the optoelectronic properties of materials adopting the first and second of these structure types,^25−29^ while this paper will focus on the third, the *A*_2_*M*O_3_Cu*Ch* type.

In the *A*_2_*M*O_3_Cu*Ch* structure type, the *anti-litharge* chalcogenide layer is separated by an oxide layer that can itself be viewed as two single-layer fragments of the *perovskite* structure whose *M*O_5_ polyhedra are separated by an *A*O *rock-salt* layer. This structure is closely related to the *A*_3_*M*_2_O_5_Cu_2_*Ch*_2_ structure type, where the *anti-litharge* chalcogenide layer is separated by a double-layer fragment of the *perovskite* structure with apex sharing of *M*O_5_ polyhedra. For reference, the relationship between the *A*_2_*M*O_3_Cu*Ch* structure and the *A*_3_*M*_2_O_5_Cu_2_*Ch*_2_ structure can be more readily expressed with doubling of the empirical formula from *A*_2_*M*O_3_Cu*Ch* to *A*_4_*M*_2_O_6_Cu_2_*Ch*_2_. As, from a compositional point of view, the only difference is the removal of the *A*O layer, then the two phases can be considered to be in a thermodynamic competition or equilibrium, as the same combination of ions can adopt either structure type without change to the oxidation state of any of the elements.^23^ There is some evidence that larger *A*^2+^ and *M*^3+^ cations favor the *A*_3_*M*_2_O_5_Cu_2_*Ch*_2_ structure, whereas smaller cations favor *A*_2_*M*O_3_Cu*Ch*,^25^ although there are a number of ion sets which can form either, if suitable precursor ratios are used (i.e., with the addition or exclusion the additional molar equivalent of the *A*O precursor), for example, both Sr_3_*M*_2_O_5_Cu_2_S_2_ and Sr_2_*M*O_3_CuS can form where *M* is Fe or Sc.^19,30,31^

In this paper, we will discuss our systematic experimental review and density functional theory (DFT) modeling of the optical and electronic properties of six Sr_2_*M*O_3_Cu*Ch* phases, where *M* = Ga, Sc, and In, with both copper sulfide and copper selenide layers, to assess their potential as wide band gap *p*-type conductors. We will also report on our attempts to synthesize their “triple layer” Sr_3_*M*_2_O_5_Cu_2_*Ch*_2_ equivalents, where we will show that this is not possible for the gallium- and indium-containing phases, where only the Sr_2_*M*O_3_Cu*Ch* structure is stable and there are no equivalent Sr_3_*M*_2_O_5_Cu_2_*Ch*_2_ phases accessible using conventional synthetic routes. Our analysis of the six Sr_2_*M*O_3_Cu*Ch* includes synthesis and characterization of the pristine materials, and also samples modified with substitutional doping of the Sr^2+^ sites with Na^+^ or K^+^ ions to introduce holes. The doping of two of these, Sr_2_GaO_3_CuS and Sr_2_InO_3_CuS, has been considered previously, where they have been assessed for their conductivity with a 5% substitution of sodium ions for strontium ions,^12^ which in this paper we extend to include assessment of potassium as a dopant. Sr_2_ScO_3_CuS, Sr_2_ScO_3_CuSe, and Sr_2_InO_3_CuS have also been identified previously,^31−33^ but we will provide a detailed assessment of their conductivity which has not previously been reported. The final compound Sr_2_GaO_3_CuSe is novel to this work, with crystal structure, band structure, and band gap reported for the first time.

Overall, we will provide evaluation of the structures, band gaps, and transport properties, from a self-consistent data set of six key materials containing the *p*-type conducting copper chalcogenide layer and adopting the Sr_2_*M*O_3_Cu*Ch* structure, and introduce alkali metal dopants to form *A*_0.05_Sr_1.95_*M*O_3_Cu*Ch*, (*A* = Na or K). We will show that from this set the most conductive doped sample is K_0.05_Sr_1.95_GaO_3_CuSe for a partially transparent material, or K_0.05_Sr_1.95_ScO_3_CuSe if full visible light transparency is required. We will also show how the geometry of the copper chalcogenide layer controls the transport properties, providing insights for future research into layered oxychalcogenides as high-mobility conductors.

## Experimental Methods

### Materials Synthesis

The six samples of layered oxychalcogenides with target compositions of Sr_2_*M*O_3_Cu*Ch*, where *M* = Ga, Sc, or In and *Ch* = S or Se, were prepared on a 0.5 g scale by direct reaction, at elevated temperature, of binary oxide and chalcogenide solid-state precursors. All precursors and products were manipulated and stored in a nitrogen-filled glovebox. As summarized in eq 1, appropriate quantities of SrO, Sr*Ch*, *M*_2_O_3_, and Cu_2_*Ch* were weighed to provide a 3:1:1:1 molar ratio and then ground together in a pestle and mortar. The mixed powder was then placed in an alumina crucible, with the crucible itself placed in a 16 mm fused silica tube which was sealed under dynamic vacuum to create an evacuated reaction ampule. The alumina crucible was used to prevent the reaction that would otherwise occur between the strontium-containing compounds and the silica ampule to produce undesired strontium silicate impurities. This sealed reaction ampule was then heated to 500 °C for 12 h in a muffle furnace, before being opened in the glovebox to recover the powder. The cycle of heating the sample in an evacuated silica ampule was repeated twice more, but with the additional step of pressing the powder into a pellet under 0.74 GPa of pressure prior to placing it in the alumina crucible. These two final heating cycles were for 12 h at temperatures between 600 and 850 °C, with the exact temperature optimized for each sample.
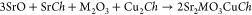
1

The precursors SrO (Fisher Scientific, 99.5%), Ga_2_O_3_ (Fisher Scientific, 99.99%), Sc_2_O_3_ (Sigma-Aldrich, 99.995%), In_2_O_3_ (Alfa Aesar, 99.998%), SrS (Fisher Scientific, 99.9%), Cu_2_Se (Alfa Aesar 99.5%), and Cu_2_S (Alfa Aesar 99.5%) were purchased and used as provided. SrSe was not available commercially, and so it was synthesized in a separate preparation from the reduction of SrSeO_4_. The SrSeO_4_ precursor was precipitated from aqueous solutions of SrCl_2_·6H_2_O (1 M, 25 mL, 99% Alfa Aesar) and Na_2_SeO_4_ (1 M, 25 mL, 99.8% Alfa Aesar) after 10 min of stirring. The SrSeO_4_ product was washed with deionized water and vacuum-filtered to remove residual NaCl. The white precipitate of SrSeO_4_ was placed in a drying oven overnight, with confirmation of purity by X-ray diffraction. The final stage was reduction of the SrSeO_4_ under a flow of 5% H_2_ in N_2_ at 650 °C in a tube furnace for 12 h. This yielded yellow SrSe, the purity of which was confirmed by using X-ray diffraction.

### Characterization

X-ray diffraction data were collected using a Bruker D2 powder diffractometer equipped with a copper K_α_ X-ray source and a *lynx eye* detector. Patterns were collected with a 2θ range of 10° < 2θ < 100° using a 0.01° step size and a minimum 3 h collection time. The powder diffraction data were analyzed using the Rietveld refinement approach with the GSAS-II software package.^34^

For optical band gap measurements, diffuse reflectance spectra were collected using a PerkinElmer Lambda 750S instrument equipped with an integrating sphere across a spectral range of 300–850 nm. Reflectance data were analyzed using the method outlined by Poeppelmeier.^35^

To determine the sample conductivity, sintered pellets were prepared. This was achieved by pressing approximately 0.15 g of each sample in an 8 mm die under 0.4 GPa of pressure to form the pellets, before sealing the pellets in silica ampules in alumina crucibles and annealing at the previously determined optimum temperature for each sample. For the scandium-containing samples, an alternative “flash” annealing process was developed, where they were placed in a furnace at 800 °C for 30 min. Once annealed, the density of the pellets was determined, and then contact points were painted onto one face of the pellet in an inverse cruciform pattern using conductive silver paint (Farnell, SCP26G). The pellets were then placed in an Ecopia SPCB-01 four-point probe spring clip mounting board attached to a Keithley DM6500 multimeter to collect a series of four-point resistance measurements to allow determination of sample conductivity using the van der Pauw method.^36^

### Computational Methodology

First-principles calculations were performed using Kohn–Sham DFT^37^ with the projector augmented wave (PAW) method^38^ as implemented in the Viena *ab initio* simulation package (VASP).^39,40^ We used the Heyd–Scuseria–Ernzerhof functional HSE06, including 25% of Hartree–Fock exact exchange^41,42^ to relax the structure and compute the electronic properties. The forces on each atom were minimized to below 0.01 eV/Å, the kinetic energy cutoff was set to 520 eV and the *k*-point grid was fixed to 6 × 6 × 2. The Sumo code^43^ was used to plot the electronic band structures and calculate the band gap. The effective conductivity masses, as defined by Gibbs et al.,^45^ and the conductivity were calculated by solving the linearized Boltzmann transport equation under the constant relaxation time (CRT) approximation using the Amset code^44^ with a typical CRT value of 10^–14^ s,^46^ and a uniform band structure calculation with *k*-point grid of 12 × 12 × 3 for each material. The effective electron and hole masses were computed at 300 K with a carrier concentration of 10^18^ cm^–3^, while a high carrier concentration of 10^21^ cm^–3^ was used to estimate the conductivity at 300 K.

## Results and Discussion

We have carried out a combined computational and synthetic investigation of the optoelectronic properties of six layered oxychalcogenides adopting the *Sr*_2_*CuGa(SO*_3_*)* structure in the *P*4/*nmm* symmetry, with composition Sr_2_*M*O_3_Cu*Ch*, where M = Ga, Sc, or In and *Ch* = S or Se. The majority of these have been reported previously, except for Sr_2_GaO_3_CuSe which is a novel composition reported here for the first time. Of the remaining five, Sr_2_GaO_3_CuS, Sr_2_ScO_3_CuS, Sr_2_InO_3_CuS, and Sr_2_InO_3_CuSe have been reported with full structural details,^22,31,33^ while Sr_2_ScO_3_CuSe has only been confirmed by indexing and refinement of lattice parameter values.^30,32^ Various optical and electronic properties have also been partially reported for these five compositions.^12,33,47^ In this work we have synthesized all six members of the isostructural series via conventional ceramic synthesis, with a purity of at least 95%, and are able to report full structural refinements, optical properties, and calculated band gaps for all of them *as a self-consistent set*. We have also measured the conductivity of these materials and their potassium- and sodium-doped equivalents, *A*_0.05_Sr_1.95_*M*O_3_Cu*Ch*, (*A* = Na or K). We find that all six materials are air-stable on a time scale of at least several weeks.

### Structure Determination

**Figure 1:**
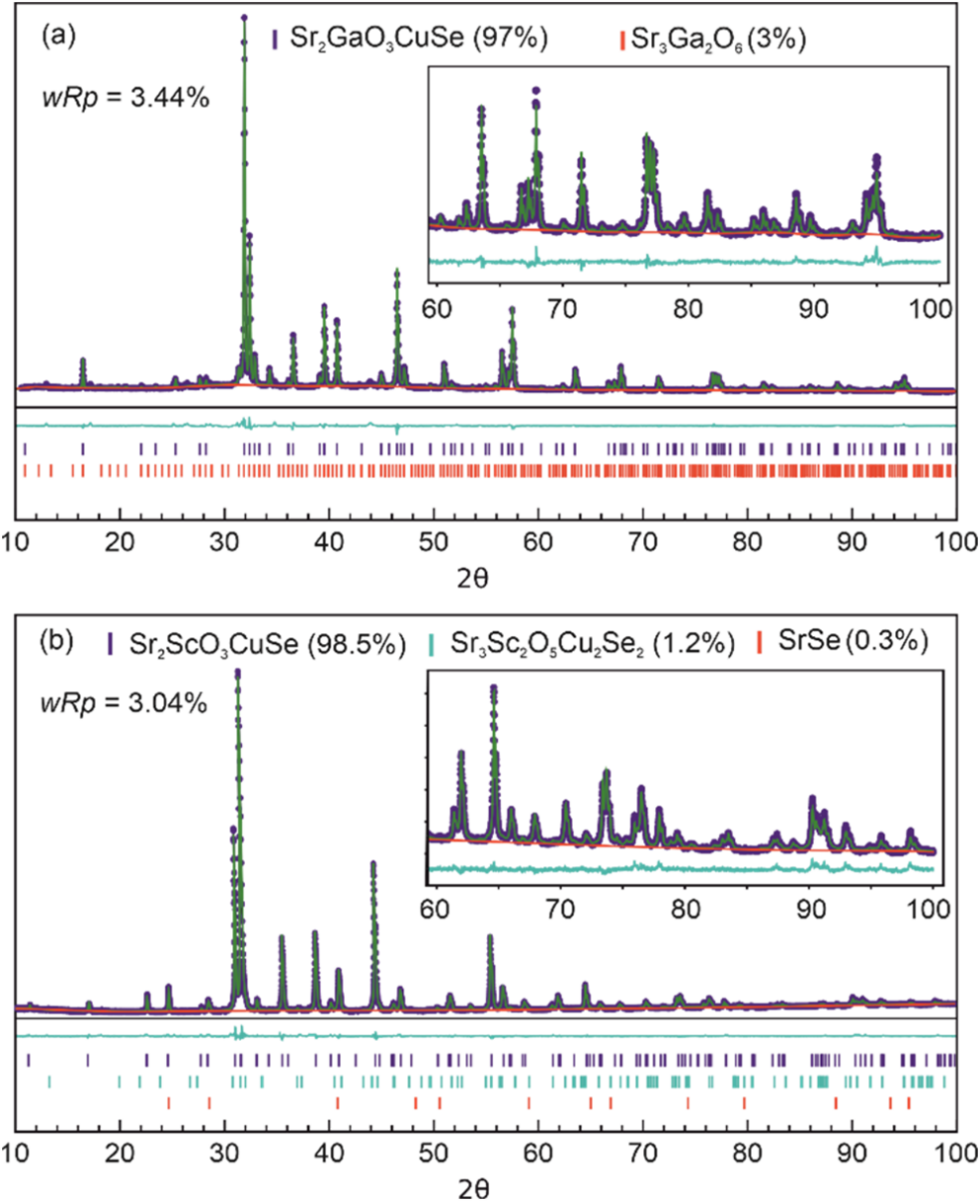
Powder X-ray diffraction data and Rietveld refinement fits of the Sr_2_*M*O_3_Cu*Ch* samples using the *P*4/*nmm* structure as the principal phase, with purity by mass of the main and secondary phases shown. Insets: (a) Sr_2_GaO_3_CuSe and (b) Sr_2_ScO_3_CuSe. In each plot, the data are shown with blue circles, the background by a red line, and Rietveld fit by a green line. The tick marks indicate the position of the Bragg reflections of the main phase (blue) and any secondary phases (cyan and red). The cyan trace below the diffraction data is the difference curve.

Analysis of the structures was carried out by Rietveld refinement against powder X-ray diffraction data, and from this, we identified that Sr_2_GaO_3_CuSe crystallizes in the *P*4/*nmm* space group with the same structure type adopted by the rest of the series. For each of the refinements discussed below, the structural model identified for Sr_2_GaO_3_CuS (ICSD 83630) was used as an initial reference model, with appropriate ion substitutions.^22^ For each refinement, the lattice parameters, atomic positions, and isotropic displacement parameters were refined to optimize the structural model. To effectively model the peak shape and profile so that reliable peak intensity information could be determined, the background was refined alongside the uniaxial sample size and strain parameters, with the [001] axis as the unique direction. The instrumental profile parameters were not refined, but instead fixed with values derived from refinement of diffraction data collected on a highly crystalline sample of LaB_6_. Where impurity peaks were found, these were modeled using the standard crystal structures identified from the ICSD, with lattice, particle size and strain, and phase fraction parameters refined. For all six compounds, we were able to identify conditions where the target phase was synthesized with at least 95% purity, as detailed for each sample below. X-ray diffraction data and Rietveld refinement fits for the two full structural refinements reported here for the first time (Sr_2_GaO_3_CuSe and Sr_2_ScO_3_CuSe) can be found in Figure 1, and the summaries of the refinement results of all of the compounds in Table 1. Full structural details can be found for each material in the ESI, and in cases where a structural model has previously been published, a comparison between our model and the prior model.

**Table 1:** Summary of Results of Rietveld Refinements for the Sr_2_*M*O_3_Cu*Ch* Samples^a^

	Sr_2_GaO_3_CuS*	Sr_2_GaO_3_CuSe	Sr_2_ScO_3_CuS*	Sr_2_ScO_3_CuSe†	Sr_2_InO_3_CuS*	Sr_2_InO_3_CuSe*
lattice parameter a/Å	3.86403(1)	3.89988(4)	4.04780(4)	4.07670(2)	4.09239(2)	4.12638(4)
lattice parameter c/Å	15.74911(6)	16.09379(17)	15.37012(20)	15.7084(1)	15.5281(1)	15.8211(2)
volume/Å^3^	235.145(2)	244.771(6)	251.834(5)	261.066(3)	260.060(3)	269.386(6)
data points	8887	4444	8985	8887	8887	8887
reflections (main phase)	104	108	111	113	111	115
parameters	46	48	46	37	43	51
purity	99.1 wt %	97.0 wt %	98.0 wt %	98.6 wt %	95.2 wt %	96.5 wt %
wRp	3.16%	3.30%	2.62%	3.23%	2.81%	4.01%
*R*_f_^2^	1.81%	2.70%	2.48%	3.02%	1.86%	4.15%
GoF	2.28	3.72	1.61	2.25	2.09	3.07
Sr1 (0.75, 0.75, *z*)	0.18435(5)	0.1931(1)	0.17947(5)	0.18840(9)	0.17706(5)	0.1848(2)
Sr2 (0.75, 0.75, *z*)	0.41401(5)	0.4160(1)	0.41286(5)	0.41500(7)	0.41351(5)	0.4158(1)
*M*, (0.25, 0.25, *z*)	Ga, 0.3121(1)	Ga, 0.3167(1)	Sc, 0.3025(1)	Sc, 0.3073(2)	In, 0.30135(5)	In, 0.3055(2)
O1 (0.25, 0.75, *z*)	0.2894(2)	0.2923(4)	0.2822(2)	0.2880(2)	0.2784(2)	0.2843(4)
O2 (0.25, 0.25, *z*)	0.4298(2)	0.4334(6)	0.4305(3)	0.4314(3)	0.4401(3)	0.4397(7)
Cu (0.25, 0.75, *z*)	0	0	0	0	0	0
*Ch*, (0.25, 0.25, *z*)	S, 0.0941(2)	Se, 0.0979(1)	S, 0.0883(1)	Se, 0.09413(9)	S, 0.0864(1)	Se, 0.0930(2)

a For samples marked with an asterisk (*), structural data has been reported previously, but the model shown here is determined from our new data to provide a self-consistent comparison. The sample marked with a dagger (†) has been reported before, but only with the lattice parameter data given.

#### Sr_2_GaO_3_CuS

This phase and its structure have been reported previously with a synthesis temperature of 950 °C,^22^ alongside measurements of its conductivity after sodium doping.^12^ The synthesis is repeated in this work, as with the others below, to allow for a self-consistent comparison between all members of the isostructural series. We found the optimum synthesis temperature to maximize sample purity to be 700 °C, lower than the previously reported value of 950 °C. The Rietveld refinement fit to the XRD data (wRp = 3.16%) produced a model with Sr_2_GaO_3_CuS present at 99.1 wt % with the impurities being small amounts of SrS (0.3 wt %) and Sr_3_Ga_2_O_6_ (0.6 wt %).

#### Sr_2_GaO_3_CuSe

This material is reported here for the first time, and we find that the optimal synthesis temperature is 830 °C. This yields a sample which analysis of the powder X-ray diffraction data by Rietveld refinement identifies as 97.0 wt % Sr_2_GaO_3_CuSe with the remaining 3.0 wt % being identified as Sr_3_Ga_2_O_6_. The wRp fit parameter for the refinement was found to be 3.30%. The diffraction data and refinement fit are listed in Figure 1(a).

#### Sr_2_ScO_3_CuS

This material has been reported previously with a synthesis temperature of 750 °C alongside DFT modeling of the band structure, but with no analysis of the conductivity.^31^ It was found that at a higher synthesis temperature of 900 °C the competing Sr_3_Sc_2_O_5_Cu_2_S_2_ phase becomes dominant based on analysis of the diffraction pattern, indicating a temperature-dependent equilibrium between the Sr_3_Sc_2_O_5_Cu_2_S_2_ and Sr_2_ScO_3_CuS structures. The full structural details of Sr_2_ScO_3_CuS were provided in the prior literature report, although there were unidentified impurities in the diffraction data.^31^ In our repeat of this synthesis with multiple regrind and reheat cycles, we find increasing amounts of the competitor phase Sr_3_Sc_2_O_5_Cu_2_S_2_ appearing in the sample at subsequent annealing steps at temperatures as low as 650 °C, alongside the dominance of this phase at higher temperatures, suggesting that the Sr_3_Sc_2_O_5_Cu_2_S_2_ structure is more thermodynamically favorable over the Sr_2_ScO_3_CuS structure for this combination of elements. Our work indicates that the optimum temperature to isolate the Sr_2_ScO_3_CuS composition exclusively is 600 °C, where after repeated heat cycles we achieve a sample purity of 98 wt %, with small amounts of SrS (1.7 wt %) and Cu (0.3 wt %) present as impurities and with a wRp fit to the diffraction data of 2.62%.

#### Sr_2_ScO_3_CuSe

Prior work on this material used a synthesis temperature of 650 °C with the authors confirming the formation of the target phase based on indexing of Bragg peaks and calculation of lattice parameters, but no structural refinement was carried out.^32^ From our experiments, we confirm that 650 °C is the optimal synthesis temperature for Sr_2_ScO_3_CuSe, as at higher temperatures the competing Sr_3_Sc_2_O_5_Cu_2_Se_2_ structure becomes increasingly dominant. For example, based on analysis of the XRD data, we find a 50:50 mixture of the Sr_3_Sc_2_O_5_Cu_2_Se_2_ and Sr_2_ScO_3_CuSe phases in samples prepared at 900 °C, increasing to 67% Sr_3_Sc_2_O_5_Cu_2_Se_2_ for samples annealed at 935 °C, implying that Sr_3_Sc_2_O_5_Cu_2_Se_2_ is the more thermodynamically favored phase of the two, similar to the case found for the sulfide analogue discussed above. At the lower, optimal synthesis temperature of 650 °C we have been able to refine a structural model for Sr_2_ScO_3_CuSe using Rietveld refinement to the resulting XRD data, Figure 1(b), with a wRp fit parameter of 3.04%. From this, we find a sample purity of Sr_2_ScO_3_CuSe of 98.5 wt %, with only a small amount of Sr_3_Sc_2_O_5_Cu_2_Se_2_ detected (1.2 wt %), and a minor amount of residual starting material, SrSe (0.3 wt %).

#### Sr_2_InO_3_CuS

This compound has been previously synthesized at temperatures between 750 and 950 °C, providing data of sufficient quality to refine the structure,^33^ but often with significant impurities of strontium sulfide and strontium indium oxides.^30^ Modeling of its band structure has been conducted using DFT methods,^33,47^ and direct measurement of conductivity; however, the impurities present may have impacted the values reported.^12^ We find that the optimum temperature for synthesis is 800 °C; however, like previous authors, we find SrS (0.8 wt %) and SrIn_2_O_4_ (4.0 wt %) as impurities with the main phase, Sr_2_InO_3_CuS, present at 95.2 wt % in our model refined against the XRD data.

#### Sr_2_InO_3_CuSe

This material has been reported previously by He et al. with a synthesis temperature of 750 °C yielding a sample with a purity of 96.2 wt % with SrIn_2_O_4_ and SrSe as impurities. These authors also measured the band gap and determined the band structure from DFT.^33^ In our experiments we find that the optimal temperature for the synthesis is slightly lower at 700 °C, which provides a sample with 96.5 wt % purity based on refinement against XRD data, with the same two minor impurities remaining: SrSe (1.5 wt %), and SrIn_2_O_4_ (2.0 wt %). The overall wRp fit parameter was found to be 4.01%.

### Structural Details and Lattice Parameter Trends

**Figure 2:**
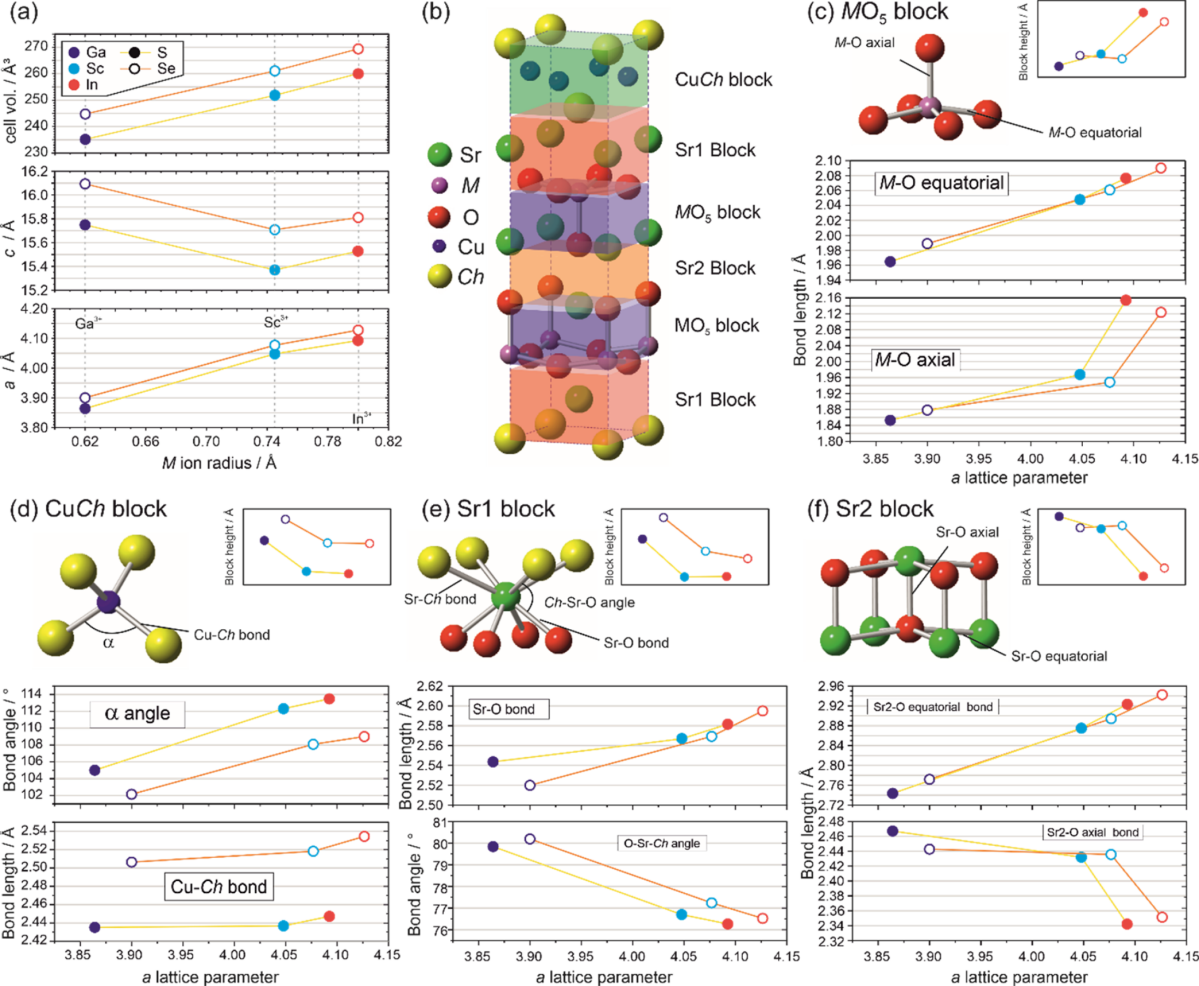
Summary of key bond lengths, angles, structural features, and lattice parameters of the Sr_2_*M*O_3_Cu*Ch* samples. All data were taken from the refinements against PXRD data collected for this work. Data associated with gallium-containing samples are shown in dark blue, scandium in light blue, and indium in red. Sulfides are shown with filled symbols and are connected with a yellow line, selenides with empty symbols connected with an orange line. (a) Plot of cell volume and lattice parameters as a function of the size of the *M*^3+^ ion. (b) Schematic of the general unit cell of Sr_2_*M*O_3_Cu*Ch*, showing the key structural “blocks”. (c–f) Key bond lengths and angles for the coordination environment of *M*, Cu, Sr1, and Sr2 sites, respectively, representing each of the key structural blocks, with the inset showing the trend in block height.

Figure 2 summarizes the key bond lengths, angles, and structural relationships in the Sr_2_*M*O_3_Cu*Ch* series. In Figure 2(a), the lattice parameters are plotted as a function of the ionic radius of the *M*^3+^ ion, and from these, we can see a series of consistent trends with isovalent substitution at both the *M* site and the chalcogenide site. As expected, the materials containing the copper selenide layer have larger unit cells compared to the equivalent copper sulfide-containing materials, with increases in both the *a* and *c* lattice parameters leading to a cell volume increase of 3.8(3)%. The observations for isovalent exchange on the *M* site with an increasing ionic radius are a little more complex. The overall cell volume and the *a* lattice parameter both increase in size as a function of the radius of the *M*^3+^ ion within both the sulfide and selenide series, as would be expected. However, in both series, there is a surprising decrease in the *c* lattice parameter with exchange of gallium for the larger scandium ion, before a slight increase with the replacement of scandium by indium. This leads to the anomaly that the gallium-containing compound has the largest *c* lattice parameter within each series, despite gallium being the smallest *M*^3+^ ion under investigation. In order to understand this observation, it is necessary to consider the structure as a series of layers or “blocks” associated with the coordination environment of the copper, strontium, and *M* transition metal ions, as shown in Figure 2(b).

As the trends across both the sulfide and selenide phases are similar, we can use the sulfides to illustrate the detailed effect of substitution on the *M* site by considering the sequence of compounds Sr_2_GaO_3_CuS - Sr_2_ScO_3_CuS - Sr_2_InO_3_CuS. Unsurprisingly the *M*-O equatorial and axial bonds increase in length with the increasing radius of *M*^3+^ ion, driving the expansion of the *a* lattice parameter and increasing the height of the *M*O_5_ block, Figure 2(c). In contrast, the response of all of the other “blocks” to the large increase of the cell in the basal direction found in Sr_2_ScO_3_CuS compared to Sr_2_GaO_3_CuS is to decrease in height. In the copper sulfide block, the expansion of the lattice in the basal direction leads to a reduction in block height in the [001] direction through a shift in the geometry, with the α angle opening up by 7.3°, consistent with the widely reported geometric flexibility of the copper chalcogenide layers.^24^ For the Sr1 interlayer site, the Sr–S and Sr–O bonds both increase slightly in length (0.10 and 0.02 Å), but there is also a change in geometry, a decrease in the S–Sr–O angle of 3.2°, leading again to an overall reduction in height in the [001] direction. The final “block” is the rock salt layer with Sr2 site, which also decreases in height, accounted for by a decrease in the Sr2–O_ax_ bond length of 0.05 Å. Overall, we can see that as the lattice expands significantly in the basal direction from Sr_2_GaO_3_CuS to Sr_2_ScO_3_CuS, three of the four “blocks” decrease in the [001] direction in response, which can be rationalized as their geometry shifting to minimize changes in bond length, leading to the overall decrease in the *c* lattice parameter with the replacement of gallium by scandium. For the transition between Sr_2_ScO_3_CuS and Sr_2_InO_3_CuS, the geometry of the *M*O_5_ pyramid expands more in the axial direction than the equatorial so that the *M*O_5_ block contributes to the overall increase in the *c* lattice parameter observed, whereas with the smaller increase in the *a* lattice parameter, the relative decrease in the height of remaining “reactive” three blocks is much smaller—although still dictated by the change in the *a* lattice parameter, as discussed above.

In terms of overall trends in the structural details, of particular interest is the copper chalcogenide geometry, as it is this layer that controls the *p*-type conductivity of this class of material. In Figure 2(d), we can see that the copper chalcogenide bond lengths remain relatively unchanged within each series regardless of the radius of the *M*^3+^ ion or the consequent *a* lattice parameter. Instead, the geometry varies through the *Ch*-Cu-*Ch* angle, which changes smoothly as a function of the size of the *M*^3+^ ion, within each series, such that it is possible to “control” this angle, ultimately as a function of the composition of the phase, covering a range of 105.0–113.5° in the sulfide-containing materials and 102.2–109.0° in the selenide-containing materials.

### Optical and Electronic Properties and Band Gap Trends

**Figure 3:**
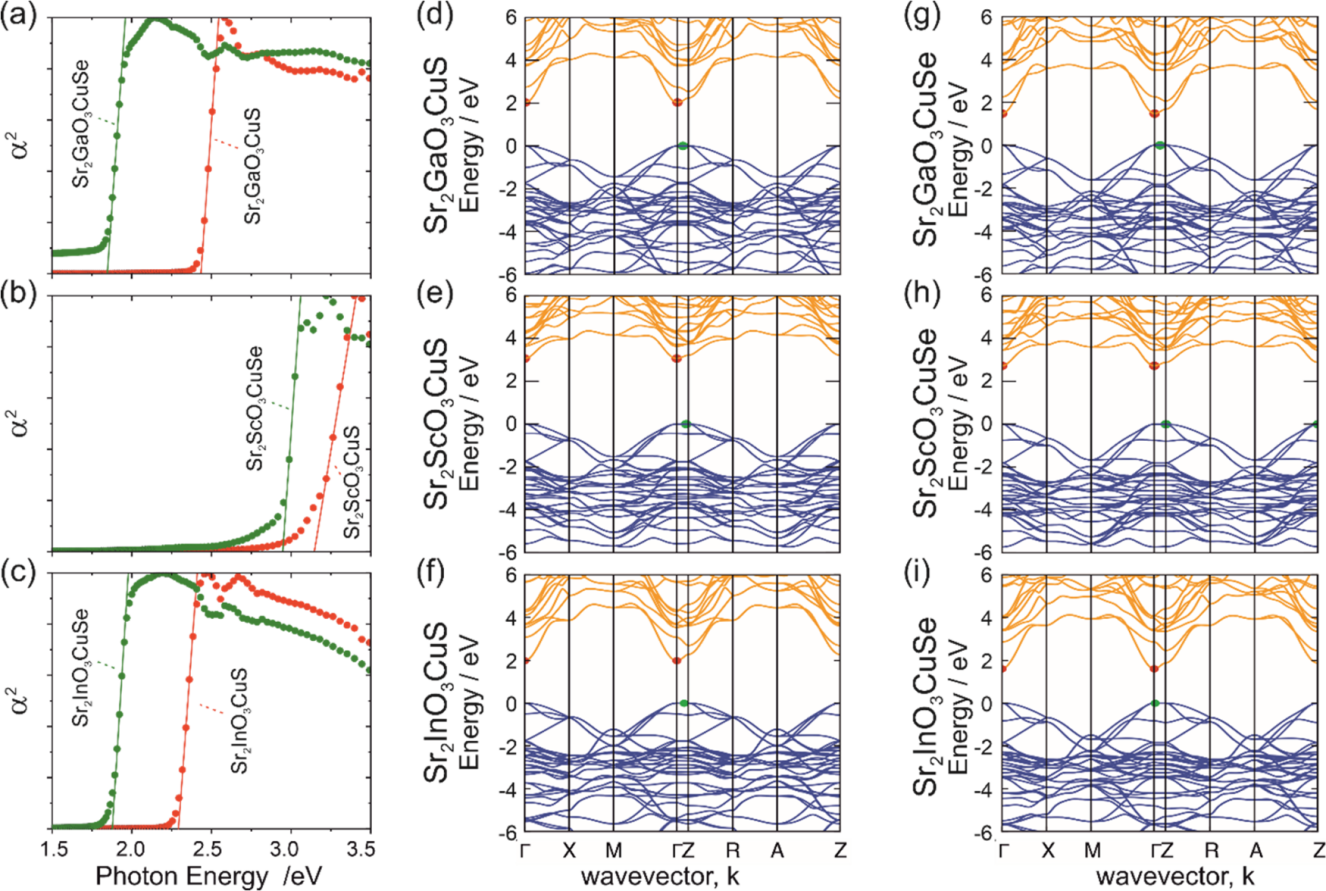
(a–c) Plots of the square of the absorption coefficient (α^2^) as a function of photon energy for the six Sr_2_*M*O_3_Cu*Ch* phases, allowing derivation of the band gap by plotting tangents to the absorption edge. Oxysulfides are shown in red, oxyselenides in green. (d–i) Electronic band structures of the layered oxychalcogenides, calculated using DFT. Valence bands are shown in blue, conduction band in orange, with VBM and conduction band minimum (CBM) shown as green and red points, respectively.

Diffuse UV–vis reflectance measurements were collected and converted to the approximate sample absorption coefficient using the Kubelka–Munk function,^48^ to determine the optical band gaps of the samples using the method outlined by Poeppelmeier.^35^ This is more appropriate than the commonly used “Tauc method”,^49^ as the Poeppelmeier method is optimized for crystalline, nondegenerate semiconductors, rather than the amorphous materials which Tauc investigated. For the Poeppelmeier method, the square of the absorption coefficient (α) is plotted as a function of the photon energy to reveal the absorption edge, as shown in Figure 3(a–c), and then, a tangent is taken from this edge to the abscissa to provide the optical band gap value. For our novel samples, this approach gave band gap values of 1.86 eV for Sr_2_GaO_3_CuSe and 1.88 eV for Sr_2_InO_3_CuSe. The four previously reported materials were also analyzed to give directly comparable data, with band gaps ranging from 2.29 to 3.17 eV as detailed in Table 2. Our values match those previously reported in the literature to within ±0.06 eV, with the exception of Sr_2_GaO_3_CuS, where we determine a band gap of 2.43 eV compared to the absorption edge measurement of 480 nm (equivalent to 2.58 eV) reported by Ueda et al. However, this literature value was determined from a simple inspection of the optical spectrum and was designed to give only an approximate indication of the visible light absorption edge rather than an accurate measure of the band gap. The computationally predicted band gaps are also reported in Table 2, which in each case slightly underpredicts the experimental value by between 0.1 and 0.4 eV. However, the same overall trends in band gap values are found in both the experimental and computational measurements.

**Table 2:** Summary of Experimental Band Gap, and DFT Modeled Optical and Electronic Properties Including Band Gap and Type, Electron and Hole Masses, and *n*-Type and *p*-Type Conductivity Assuming Heavy Doping

	band gap/eV			effective carrier mass			
composition	*E*_g_ (opt.)	*E*_g_ (comp.)	type	m* (hole)	m* (electron)	*p*-type @10^21^ cm^3^/ S cm^–1^	*n*-type @10^21^ cm^3^/ S cm^–1^
Sr_2_GaO_3_CuS	2.43	2.03	indirect	0.69	0.25	1850	4630
Sr_2_GaO_3_CuSe	1.86	1.48	indirect	0.56	0.24	2540	4670
Sr_2_ScO_3_CuS	3.17	3.07	indirect	0.86	0.36	1790	2330
Sr_2_ScO_3_CuSe	2.95	2.73	indirect	0.63	0.31	2210	2390
Sr_2_InO_3_CuS	2.29	1.98	indirect	0.95	0.24	1930	4920
Sr_2_InO_3_CuSe	1.88	1.61	indirect	0.57	0.21	2410	4890

Computational methods were used to determine the band structure of each compound using hybrid DFT calculations, and plots of the *E* vs *K* diagrams resulting from these can be found in Figure 3(d–i). The band structure diagrams show that all of the Sr_2_*M*O_3_Cu*Ch* samples have slightly indirect band gaps, with the conduction band minimum (CBM) at the Γ point and the valence band maximum (VBM) at the *Z* point or on the Γ-*Z* path. However, all of these are close to being direct gaps, and in particular, the band gap of Sr_2_InO_3_CuS, although formally indirect, has the VBM sitting extremely close to the Γ point, directly below the CBM. Analysis of the orbital composition finds that for all compounds, the valence band maximum (VBM) arises primarily from the overlap of the chalcogenide p orbitals. This explains the common feature that the selenide compounds have a smaller band gap than the equivalent sulfides, as the greater electronegativity of sulfur shifts the VBM lower in energy when compared to the equivalent selenide-containing compound, opening the band gap. For the scandium-containing phases, the CBM is composed of Ba 5d and Cu 4s states, relatively high in energy giving both Sr_2_ScO_3_CuS and Sr_2_ScO_3_CuSe UV absorbing band gaps with energies greater than 2.9 eV. When scandium is replaced by gallium or indium, these more electropositive post-transition metals have lower 5s orbitals which then dominate the CBM leading to a reduction in the band gap to 2.3–2.4 eV for the sulfides and 1.8–1.9 eV for the selenides, such that they all absorb a portion of the visible light spectrum. There is only a small difference in the indium and gallium electronegativity values, explaining the similar band gap for their respective sulfides and selenides.

The transport properties of the compounds were also determined from DFT calculations using Boltzmann transport theory. These modeled transport properties are summarized in Table 2. Effective hole and electron masses were calculated by assuming a carrier concentration of 10^18^ cm^–3^, while the maximum in-plane *n*-type and *p*-type conductivity values were calculated by assuming heavy doping values of 10^21^ cm^–3^. The models predicted good *p*-type transport for the copper sulfide phases, with conductivity values 1.8 × 10^3^ and 1.9 × 10^3^ S cm^–1^ and corresponding hole masses of 0.7–1.0 m_e_. The copper selenides all had better-predicted transport properties with conductivities greater than 2.2 × 10^3^ S cm^–1^ and hole masses of approximately 0.6 m_e_, with the best-predicted values for Sr_2_GaO_3_CuSe, with a hole mass of 0.56 m_e_ and a conductivity of 2.5 × 10^3^ S cm^–1^. These can be placed within the context of viable transparent conductor coatings requiring conductivities of 1 × 10^3^–1 × 10^4^ S cm^–1^, indicating that all are good candidate materials based on their modeled transport properties—if the high dopant levels can be realized. Although the layered copper chalcogenides have been found to be natively *p*-type, and increasing the hole concentration has also been the focus of this and prior work on them, the modeling indicates that the six Sr_2_*M*O_3_Cu*Ch* phases could also be effective *n*-type conductors, as they show good electron mobility, with *n*-type conductivities of approximately 2.3 × 10^3^ S cm^–1^ for the scandium materials, 4.6 × 10^3^ S cm^–1^ for the gallium materials, and 4.9 × 10^3^ S cm^–1^ for the indium-containing phases. This is intriguing as it presents the possibility of using a layered copper chalcogenide with both acceptor- and donor-doped regions for the formation of transparent junctions.

Pressed 8 mm pellets of all six compounds were prepared for experimental conductivity measurements, alongside equivalents doped with sodium and potassium, with target compositions of *A*_0.05_Sr_1.95_*M*O_3_Cu*Ch* where *A* = Na, and K, yielding a total of 18 samples for comparative conductivity measurements. For the doped samples the 5 at. % alkali metal concentration was chosen based on prior work on similar layered oxychalcogenides which indicated that this is the optimum value to maximize conductivity.^29^ XRD measurements were conducted to confirm sample purity and UV–vis spectra to confirm band gaps. This data can be found in the ESI. Pellet densities were also measured and found to vary between 64 and 81%, which are typical values for cold-pressed pellets with subsequent annealing. The XRD data confirmed the formation of the target phase in each case, although typically at a reduced purity for the doped samples compared to the “pristine” undoped equivalents. The target dopant concentrations were too small to be directly confirmed by refinement against powder X-ray diffraction data, but successful doping was inferred from the effect on the conductivity and the absence of sodium or potassium-containing byproducts. For all of the samples, no difference in the band gap was found for any doped sample compared to the undoped equivalent within the error of the measurements; therefore, there is no evidence that carrier concentrations reached sufficient levels for a Moss-Burstein shift to be observed for any of the compositions. A summary of the resulting conductivity measurements, pellet densities, and sample purities can be found in Table 3, and below we discuss in detail the conductivity and effect of doping on each of the compositions in turn.

**Table 3:** Summary of the Experimental Conductivity, Density, and Purity of Cold-Pressed Pellets of Sr_2_*M*O_3_Cu*Ch* Samples, with and without Sodium or Potassium Doping

	pristine			Na_0.05_Sr_1.95_MO_3_CuCh			K_0.05_Sr_1.95_MO_3_CuCh		
parent sample	conductivity/S cm^–1^	pellet density	purity	conductivity/S cm^–1^	pellet density	purity	conductivity/S cm^–1^	pellet density	purity
Sr_2_GaO_3_CuS	2.38 × 10^–4^	74.3%	99.1%	1.61 × 10^–2^	73.5%	98.1%	2.84 × 10^–4^	77.8%	97.5%
Sr_2_ScO_3_CuS	8.48 × 10^–5^	65.0%	99.4%	2.22 × 10^–4^	64.7%	94.9%	2.05 × 10^–5^	67.3%	89.6%
Sr_2_InO_3_CuS	1.4 × 10^–5^	70.9%	95.2%	2.09 × 10^–6^	73.7%	83.1%	7.52 × 10^–6^	74.5%	84.8%
Sr_2_GaO_3_CuSe	1.54 × 10^–1^	78.0%	97.0%	1.00 × 10^–1^	76.5%	99.6%	4.59 × 10^–1^	68.4%	99.4%
Sr_2_ScO_3_CuSe	6.29 × 10^–4^	67.9%	98.5%	8.23 × 10^–3^	66.3%	90.3%	2.30 × 10^–2^	69.3%	90.3%
Sr_2_InO_3_CuSe	1.45 × 10^–3^	71.5%	96.5%	1.24 × 10^–2^	81.4%	95.2%	4.35 × 10^–3^	76.5%	86.2%

#### Sr_2_GaO_3_CuS

Synthesis and preparation of the pellets were successful for the samples, with sintering at 700 °C yielding pellet densities between 74 and 78%, the high end of the range possible using our cold-press approach. The sample purity was also high, 99.1 wt % for the undoped sample, dropping only slightly for both doped samples to 98.1 and 97.5 wt % for the sodium and potassium doped samples, respectively. In the sodium-doped sample, the impurities were SrS and Sr_3_Ga_2_O_6_, which were also found in the potassium-doped sample alongside 0.7 wt % of KCu_7_S_4_ as an additional impurity. The KCu_7_S_4_ impurity represents approximately 13 mol % of the expected potassium and is indicative of limited potassium solubility into Sr_2_GaO_3_CuS. The conductivity of undoped Sr_2_GaO_3_CuS was determined to be 2.4 × 10^–4^ S cm^–1^, with the nominally potassium-doped sample showing almost no change with a conductivity of 2.8 × 10^–4^ S cm^–1^, either due to failure to incorporate potassium into the structure, or due to poor hole formation efficiency. In contrast, the sodium doping leads to an increase in conductivity of almost 2 orders of magnitude to 1.6 × 10^–2^ S cm^–1^, and the largest increase for any of the materials investigated in this work. These results are in line with prior work on this composition, where Ueda et al. found a conductivity of 2.2 × 10^–4^ S cm^–1^ for an undoped sample Sr_2_GaO_3_CuS, and 2.4 × 10^–2^ S cm^–1^ for a sodium doped sample with nominal composition Na_0.1_Sr_1.9_GaO_3_CuS, i.e., twice the sodium concentration compared to our sample.^12^

#### Sr_2_ScO_3_CuS

Due to the existence of a competing and more stable layered chalcogenide phase, with composition Sr_3_Sc_2_O_5_Cu_2_S_2_, selection of the annealing temperature for preparation of the pellets of Sr_2_ScO_3_CuS for conductivity testing was a balance between achieving a sufficient temperature to densify the pellets while preventing conversion to the more thermodynamically stable Sr_3_Sc_2_O_5_Cu_2_S_2_ composition. Unfortunately, an optimum balance could not be achieved. The maximum temperature that we found that could be used without significant conversion to the Sr_3_Sc_2_O_5_Cu_2_S_2_ phase was 600 °C, but this was insufficient to give the necessary densification; the densities remained below 65% of the theoretically expected values. A process of flash annealing was developed, where the sample was placed in a furnace at 800 °C for 30 min; however, even under this treatment, it was still only possible to reach pellet densities of 65–67%. This allowed for a relatively pure undoped sample of 99.4 wt % purity, but for the sodium and potassium doped samples, the purity dropped to 83.1 and 90.9 wt %, with Sr_3_Sc_2_O_5_Cu_2_S_2_ being the major impurity. The pristine sample of Sr_2_ScO_3_CuS had a conductivity of 8.5 × 10^–5^ S cm^–1^. The nominally potassium-doped sample had a slightly lower conductivity of 7.5 × 10^–6^ S cm^–1^, while for the sodium-doped sample, a small increase to 2.2 × 10^–4^ S cm^–1^ was observed.

#### Sr_2_InO_3_CuS

For the indium-containing layered oxysulfide, the pellets were annealed at 800 °C, and good densities were achieved: 71% for the pristine sample and 74–75% for the sodium and potassium doped samples. However, there was a significant decline in sample purity with doping, from 95.2 wt % for the undoped sample to 83.1 and 84.8 wt % for the sodium-doped and potassium-doped samples, respectively, with the impurities being SrS, SrIn_2_O_4_, and Cu. Although there was no direct evidence of sodium or potassium-containing side phases, the high level of overall impurity indicated that there was not a ready uptake of the dopant for either sample. This was reflected in the conductivity measurements, where we found that the undoped sample of Sr_2_InO_3_CuS had a conductivity of 1.4 × 10^–5^ S cm^–1^, and this dropped for the nominally sodium and potassium doped samples to 2.1 × 10^–6^ and 7.5 × 10^–6^ S cm^–1^, respectively, providing further evidence that Sr_2_InO_3_CuS could not be effectively doped to increase its charge carrier concentration. These are the lowest conductivities among the compounds investigated in this study. Interestingly, the presence of up to 1.8 wt % metallic copper found in the nominally doped samples did not lead to any increase in the overall conductivity. Comparing our values to the literature, we find our reported conductivity for the undoped Sr_2_InO_3_CuS is significantly higher than the value first reported for a sample of this compound of 2.2 × 10^–10^ S cm^–1^,^12^ although more recently He et al. have reported 2.0 × 10^–4^ S cm^–1^.^33^ This suggests that significant variation in the baseline carrier concentration is possible in samples of Sr_2_InO_3_CuS, possibly due to small variation in precursor purity, although all of the reported values are too low to be of any interest for potential application.

#### Sr_2_GaO_3_CuSe

The pellets of doped and undoped Sr_2_GaO_3_CuSe were annealed at 700 °C, and good densities were achieved between 77 and 78%. The sample purity was also excellent, 97.0 wt % for the undoped, with values higher for the doped samples (99.6 and 99.4 wt % for sodium and potassium doped), although there was a small unidentified peak in the diffraction patterns of both doped samples which could not be assigned so that the actual purity is likely to be slightly lower. The main peak of this unidentified impurity appears in both samples at the same position (17.2°), indicating that it is unlikely to be a sodium- or potassium-containing phase. The undoped Sr_2_GaO_3_CuSe sample had the highest conductivity of the six undoped samples, at 1.54 × 10^–1^ S cm^–1^, with a slightly lower value for the nominally sodium-doped at 1.0 × 10^–1^ S cm^–1^, indicating that sodium was not effective for hole generation. In contrast, the potassium-doped sample had a higher conductivity of 4.6 × 10^–1^ S cm^–1^, making K_0.05_Sr_1.95_GaO_3_CuSe the most conductive sample identified within the overall set under investigation here.

#### Sr_2_ScO_3_CuSe

As with the sulfide analogue, the scandium-containing layered oxyselenide could not be annealed at high temperatures due to competition with the formation of Sr_3_Sc_2_O_5_Cu_2_Se_2_, so the flash anneal approach was used instead with a 30 min exposure at 800 °C. This yielded pellets with poor densities of 66–69%, with relatively high purity for the undoped sample (98.5 wt %) but much lower for the doped samples, both of which had main phase purity of 90.3 wt %. The main impurity in each case (∼8 wt %), was the Sr_3_Sc_2_O_5_Cu_2_Se_2_ competing layered chalcogenide, with SrSe and SrCO_3_ present in small amounts. The potassium-doped phase also had KCu_3_Se_2_ present, but only at 0.3 wt %, and so did not represent a significant fraction of the potassium used in the synthesis. The conductivity was found to be 6.3 × 10^–4^ S cm^–1^ for the undoped sample, with significant increases after doping to 1.2 × 10^–2^ S cm^–1^ for the sodium-doped sample, and 4.4 × 10^–3^ S cm^–1^ for the potassium-doped sample, indicating that both alkali metal dopants were effective at hole formation, but with sodium the more effective of the two.

#### Sr_2_InO_3_CuSe

For our final sample, the pellets were annealed at 700 °C, yielding good pellet density for all samples −72% for undoped Sr_2_InO_3_CuSe, and 81 and 77% for sodium doped and potassium doped, respectively. From the Rietveld analysis, the sample purity for the undoped sample was determined to be 96.5 wt %, with a similar value for the sodium-doped analogue of 95.2 wt %, but much lower for the potassium-doped sample of 86.2 wt %. SrSe and Sr_2_InO_4_ were identified as the impurities in each case, just in higher percentages in the case of the nominal K_0.05_Sr_1.95_InO_3_CuSe sample. Both sodium and potassium doping increased conductivity compared to the undoped material, with sodium doping slightly more so, indicating sodium is the more efficient hole-forming dopant for this composition, but this could also be explained by it being the purer and denser sample. The value of the conductivity for the undoped sample was found to be 1.5 × 10^–3^ S cm^–1^, increasing to 1.2 × 10^–2^ and 4.4 × 10^–3^ S cm^–1^ for the sodium- and potassium-doped samples, respectively.

### Trends in the Experimental Conductivity

An attempt to identify a definitive correlation between the experimental conductivity and the structure or composition of the samples is hampered by the additional variation in sample purity and pellet density, which were not possible to directly control but which will also have a significant effect on the transport measurements. This is particularly frustrating for the scandium-containing samples as it was not possible to use a sufficiently high temperature to densify the pellets without a catastrophic loss of purity through conversion to the respective Sr_3_Sc_2_O_5_Cu_2_*Ch*_2_ competing phase. The results of doping within the study are also less effective than those in prior work on layered oxychalcogenides. In previous work at least one of the dopants studied (typically sodium or potassium ions) would yield an increase of approximately 2 orders of magnitude,^29^ and often much higher with an astonishing increase from 2.2 × 10^–8^ S cm^–1^ in Sr_2_ZnO_2_Cu_2_S_2_ to 1.2 × 10^–1^ S cm^–1^ in Na_0.1_Sr_1.9_ZnO_2_Cu_2_S_2_.^12^ In this work, the highest increase observed is in K_0.05_Sr_1.95_GaO_3_CuS, with just under 2 orders of magnitude increase over the pristine, whereas with the optimal dopant, Sr_2_GaO_3_CuSe, Sr_2_ScO_3_CuSe, Sr_2_InO_3_CuSe, and Sr_2_ScO_3_CuS only achieve a single order of magnitude or less increase in conductivity. We also identify one sample where the doping only worsens the conductivity, Sr_2_InO_2_CuS. The reason for this remains unclear; as stated above, this could be due to a combination of varying density and purity in the doped samples or a size mismatch with the dopants for this series of compounds leading to limited uptake or increased lattice strain reducing mobility in the doped samples. Although we did not have access to the technique, spark plasma sintering might be used in the future to achieve higher pellet densities,^14^ and conductivities closer to the values predicted computationally. Given these caveats, however, we can tentatively identify some key trends within the set of 18 samples prepared for this work.

**Figure 4:**
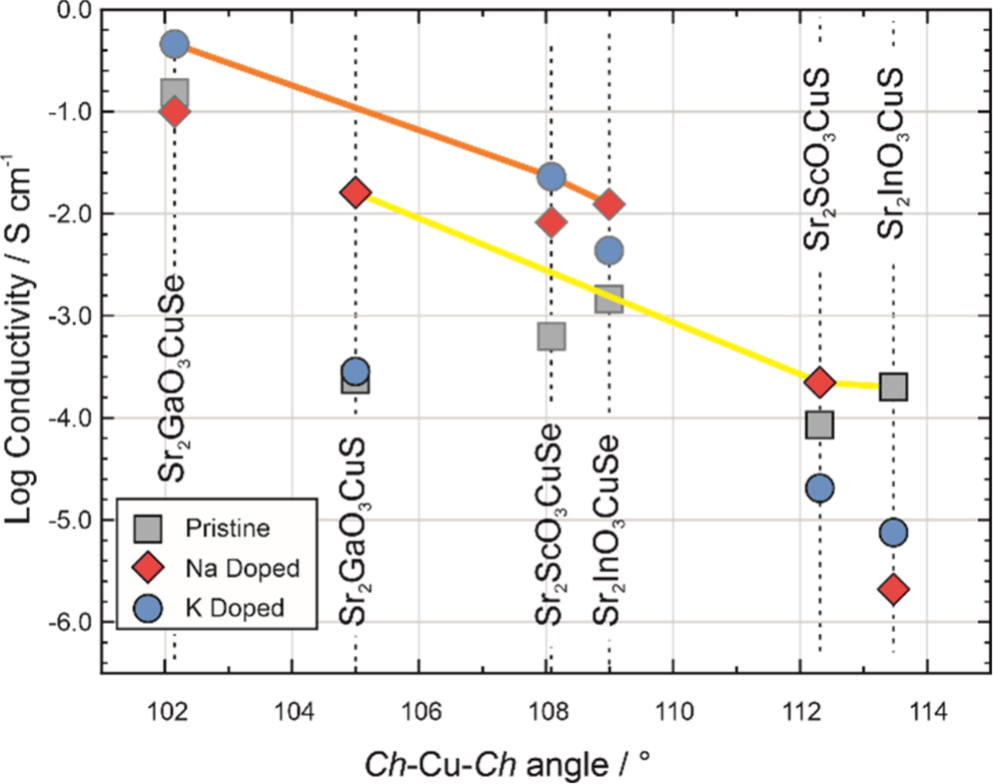
Plot of the log conductivity against copper-chalcogenide angle for the pristine samples of Sr_2_*M*O_3_Cu*Ch* shown by gray squares, with the sodium-doped Na_0.05_Sr_1.95_*M*O_3_Cu*Ch* by red diamonds and potassium-doped K_0.05_Sr_1.95_*M*O_3_Cu*Ch* in blue circles. The copper sulfide samples are highlighted by black outlines with the best conductivity of each type connected by a yellow line. The copper selenide samples are highlighted with dark gray outlines, with the most conductive of each type connected by an orange line. From this, the general trends can be seen of selenide phases having higher conductivity than sulfides and the trend for increasing conductivity with smaller bond angles. The value for undoped Sr_2_InO_3_CuS is taken from ref (33).

The primary overall observation is that the copper selenide phases are more conductive than the equivalent copper sulfides, which can be understood in terms of the greater dispersion and overlap of the selenide orbitals. Investigation of a copper telluride adopting this structure has been carried out which shows the trend continuing, with undoped Sr_2_InO_3_CuTe having a conductivity of 1.7 S cm^–1^; however, the band gap also decreases (to 1.2 eV), so Sr_2_InO_3_CuTe is not viable for applications where visible light transmission is needed.^33^ A secondary trend is that within each group of either sulfide or selenide compounds the conductivity decreases with increasing *M*^3+^ ion size in the order Ga^3+^ < Sc^3+^ < In^3+^. Among the undoped materials in our data set, Sr_2_ScO_3_CuSe breaks this trend, but if the most conductive sample of each composition is used, to mitigate the effects of density and purity, a clearer trend emerges, without the anomaly. This secondary trend can be explained by the smaller transition metal ions driving a shift in the copper layer geometry, which brings the chalcogenide ions closer to each other as the size of the cell decreases in the basal direction, leading to improved orbital overlap of the chalcogenide 3p or 4p orbitals that contribute to the VBM and hence the hole mobility. We have tried to quantify this relationship by plotting the conductivity as a function of the in-plane *Ch*-Cu-*Ch* α bond angle for each of the samples in Figure 4. From this plot, it can be seen that the smaller *Ch*-Cu-*Ch* angles correlate with higher conductivity, which we hypothesize is due to the increased chalcogenide orbital overlap in the basal direction, which is the principal direction of the conductivity in these layered materials. This observation must be taken within the context of the small sample size, but it seems to be a plausible effect, and one that could be considered in further studies, with an attempt to minimize the α angle to target materials for improved conductivity. Further work could also explore alternative doping strategies, such as substitution at the *M*^3+^ site with lower valent calcium or magnesium ions or a greater range of dopant concentrations.

### Attempt to Synthesize Competing Sr_3_*M*_2_O_5_Cu_2_*Ch*_2_ Analogues

It has been previously shown for the scandium-containing compounds, Sr_2_ScO_3_CuS and Sr_2_ScO_3_CuSe, that the same ion combinations (Sr^2+^, Sc^3+^, O^2–^, Cu^+^, and *Ch*^2–^) can also form the Sr_3_Sc_2_O_5_Cu_2_*Ch*_2_ composition if less SrO is included in the precursor mix, as shown in eq 2.^19,32^
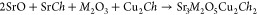
2

The ability to form the two related phases is also true for other ion combinations, for example, both Sr_3_Fe_2_O_5_Cu_2_S_2_ and Sr_2_FeO_3_CuS can be formed.^30^ In other cases it seems that only one or the other is stable, for example, Sr_2_VO_3_FeAs is stable, but Sr_3_V_2_O_5_Fe_2_As_2_ cannot be formed.^23,50^ For Sr_3_Sc_2_O_5_Cu_2_*Ch*_2_ and Sr_2_ScO_3_Cu*Ch*, even though all phases can be made, it is also the case that they are in competition, and that for the scandium materials, the Sr_3_Sc_2_O_5_Cu_2_*Ch*_2_ phases are the more stable, as even without reducing the SrO fraction in the precursor ratio, Sr_3_Sc_2_O_5_Cu_2_*Ch*_2_ is often found as an impurity in the attempted synthesis of the Sr_2_ScO_3_Cu*Ch* materials, with the amount of Sr_3_Sc_2_O_5_Cu_2_*Ch*_2_ increasing at higher synthesis temperatures.^31^ As discussed above, the optimum temperature for the formation of Sr_2_ScO_3_CuS is 600 °C, while that of Sr_2_ScO_3_CuSe is 650 °C. We find that at temperatures above this, the respective Sr_3_Sc_2_O_5_Cu_2_*Ch*_2_ phases start to appear in the samples, becoming dominant (more than 50%) above 800 °C. For samples attempted at synthesis temperatures of 900 °C, the selenide with nominal composite Sr_2_ScO_3_CuSe shows a 67% conversion to Sr_3_Sc_2_O_5_Cu_2_Se_2_, while the sulfide analogue is entirely converted to Sr_3_Sc_2_O_5_Cu_2_S_2_.

Given the existence and higher apparent stability of the scandium-containing Sr_3_Sc_2_O_5_Cu_2_*Ch*_2_ phases, we attempted the synthesis of the related indium and gallium phases, Sr_3_*M*_2_O_5_Cu_2_*Ch*_2_ where *M* = In or Ga, with a synthesis temperature of 800 °C, to test if these ion combinations can also form the alternative structure. In each case, the attempt was a failure, with no indication of the formation of a Sr_3_*M*_2_O_5_Cu_2_*Ch*_2_ structure with plausible lattice parameters, and only various binary, ternary, or Sr_2_*M*O_3_Cu*Ch* competing phases identified in the diffraction data. We also found no evidence of any impurity in the XRD patterns collected from our direct attempts at forming Sr_2_*M*O_3_Cu*Ch* reported above, whereas in the scandium-containing materials, additional peaks associated with Sr_3_Sc_2_O_5_Cu_2_*Ch*_2_ were often present when attempting a synthesis of the Sr_2_ScO_3_Cu*Ch* compounds. In summary, we find no evidence that Sr_3_*M*_2_O_5_Cu_2_*Ch*_2_ will form if *M* = Ga or In, with the Sr_2_*M*O_3_Cu*Ch* structure forming preferentially instead, despite the precursor mix being poorer in SrO. Only when *M* = Sc, can both phases be isolated, when appropriate precursor ratios are used, and with evidence that the Sr_3_*M*_2_O_5_Cu_2_*Ch*_2_ compound is more thermodynamically stable. In contrast, we have previously shown that when barium is used on the *A* site, *only* the Ba_3_*M*_2_O_5_Cu_2_*Ch*_2_ materials are formed for both selenide and sulfide phases, with no evidence of the formation of an air-stable Ba_2_*M*O_3_Cu*Ch* material being possible.^25−27^ Examples of the barium- and gallium-containing phases of either Ba_3_Ga_2_O_5_Cu_2_*Ch*_2_ or Ba_2_GaO_3_Cu*Ch* phases are not known, almost certainly due to the large size mismatch between the larger barium and much smaller gallium ions. The work on the experimental stability of all of the *A*_2_*M*O_3_Cu*Ch* and *A*_3_*M*_2_O_5_Cu_2_*Ch*_2_ phases is summarized in Table 4.

**Table 4:** Summary of the Relative Stability of the Two Possible Structure Types *A*_3_*M*_2_O_5_Cu_2_*Ch*_2_ and *A*_2_*M*O_3_Cu*Ch* Stating if either or both Can Be Formed from Appropriate Ratio of Precursors, for *A* = Sr or Ba, M = Ga, Sc or In, and *Ch* = S or Se

element combination	*A*_3_*M*_2_O_5_Cu_2_*Ch*_2_	*A*_2_*M*O_3_Cu*Ch*
Sr–Ga–O–Cu–S	X unstable	√ reported^12^
Sr–Ga–O–Cu–Se	X unstable	√ reported (this work)
Sr–Sc–O–Cu–S	√ reported^14^	√ reported^31^
Sr–Sc–O–Cu–Se	√ reported^20^	√ reported^32^
Sr–In–O–Cu–S	X unstable	√ reported^12^
Sr–In–O–Cu–Se	X unstable	√ reported^33^
Ba–Sc–O–Cu–S	√ reported^26^	X unstable
Ba–Sc–O–Cu–Se	√ reported^25^	X unstable
Ba–In–O–Cu–S	√ reported^27^	√ reported - air sensitive^33^
Ba–In–O–Cu–Se	√ reported^27^	X unstable

The clear overall correlation seems to be that a larger ion on the *A* site favors the formation of the A_3_M_2_O_5_Cu_2_Ch_2_ structure, while a smaller *A* site ion favors the formation of the *A*_2_*M*O_3_CuCh structure. This is supported by work conducted by Clarke et al.,^51,52^ who investigated mixtures of Sr/Ba, Sr/Ca, and Ba/Ca in *A*_3_Fe_2_O_5_Cu_2_Ch_2_ which contains two possible alkaline earth sites, the larger 12 coordinate intralayer site (within the oxide block), and a smaller 8 coordinate interlayer site, between the oxide and chalcogenide blocks. They find that in these mixed *A* site iron-containing phases, *A*_3_Fe_2_O_5_Cu_2_Ch_2_, the larger *A* ion preferentially occupies the 12 coordinate intralayer site. In contrast, although the *A*_2_MO_3_CuCh structure has a similar 8-coordinate interlayer site, the intra-oxide layer site is a smaller 5-coordinate site. The lack of the large 12 coordinate site and the presence of only the two “smaller” A sites, explains the preference for the A_2_MO_3_CuCh structure with compounds made using the smaller strontium ion, and the *A*_3_M_2_O_5_Cu_2_Ch_2_ structure with the larger barium ion.

## Conclusions

We have systematically investigated the structure, band gap, band structure, and transport properties of 18 doped *A*_0.05_Sr_1.95_*M*O_3_Cu*Ch* (*A* = Na or K) and undoped Sr_2_*M*O_3_Cu*Ch* compounds, identified Sr_2_GaO_3_CuSe as a novel phase and confirmed the structure of Sr_2_ScO_3_CuSe. Within the set of compounds reported here, we find that the most conductive compound is K_0.05_Sr_1.95_GaO_3_CuSe, with a measured conductivity of 0.46 S cm^–1^. Although this is an excellent conductivity for a cold-pressed pellet measurement, the band gap of 1.9 eV rules out its use in transparent conductor applications. The best conductive sample with good visible light transparency is K_0.05_Sr_1.95_ScO_3_CuSe with a band gap of 2.9 eV and a conductivity of 2.3 × 10^–2^ S cm^–1^. Although this conductivity is too low to be used in commercial applications, improvements could be achieved through sample densification. More significantly we have identified a trend indicating that the geometry of the copper layer is the controlling factor on the conductivity and that attempts at increasing the conductivity of layered copper chalcogenides should focus on minimizing the in-plane copper chalcogenide angle to increase the dispersion of the valence band and maximize the hole mobility.

## Data Availability

Data supporting this study are openly available from the University of Southampton repository at 10.5258/SOTON/D3291 and the Zenodo repository DOI: 10.5281/zenodo.13986868.

## Supporting Information Available

Section A: Rietveld refinement fits to X-ray diffraction data for all six Sr_2_*M*O_3_Cu*Ch* materials: diffraction data for the compounds (Figures S1–S6); Section B: Tables comparing our refined structural models to prior literature, where available (Tables S1–S4); Section C: Full structural details of all six Sr_2_*M*O_3_Cu*Ch* compounds, derived from samples and diffraction data prepared for this work (Tables S5–S10); summary of all of the major bond angles, lengths, and block heights (Table S11); Section D: Rietveld refinement fits and summary for sodium- and potassium-doped *A*_0.05_Sr_1.95_*M*O_3_Cu*Ch* samples (Figures S7–S18); summary of the band gaps the doped samples, and the results of their refinements (Table S12) (PDF)
