# Supplementary material for: Transport Properties of Doped Wide Band Gap Layered Oxychalcogenide Semiconductors Sr2GaO3CuCh, Sr2ScO3CuCh, and Sr2InO3CuCh (Ch = S or Se)
Source: Chem Mater. 2024 Nov 14;36(22):11326–37. doi: 10.1021/acs.chemmater.4c02760 (PMC11603606; doi:10.1021/acs.chemmater.4c02760)
Supplement: Supplementary file 1 — cm4c02760_si_001.pdf [file cm4c02760_si_001.pdf]

Supplementary Information

Transport properties of doped wide band gap layered oxychalcogenide semiconductors Sr_2GaO_3CuCh, Sr_2ScO_3CuCh and Sr_2InO_3CuCh (Ch = S or Se)

*Zahida Malik,[1] Liam Kemp,[1] Bastien F. Grosso,[2] Daniel W. Davies,[3] David O. Scanlon,[2] Geoffrey Hyett.[1]**

[1] School of Chemistry, Faculty of Engineering and Physical Sciences, Highfield Campus, University of Southampton, SO17 1BJ, UK

[2] School of Chemistry, University of Birmingham, Edgbaston, Birmingham, B15 2TT, UK

[3] Department of Chemistry, University College London, London, WC1H 0AJ, UK

Section A: Rietveld refinement fits to X-ray diffraction data for all six Sr_2MO_3CuCh materials

Section B: Tables comparing our refined structural models to prior literature, where available

Section C. Full structural details of all six Sr_2MO_3CuCh compounds, derived from samples and diffraction data prepared for this work

Section D. Rietveld refinement fits and summary for sodium and potassium doped $A_{0.05}Sr_{1.95}MO_3CuCh$ samples

Section A: Rietveld refinement fits to X-ray diffraction data for all six Sr_2MO_3CuCh materials

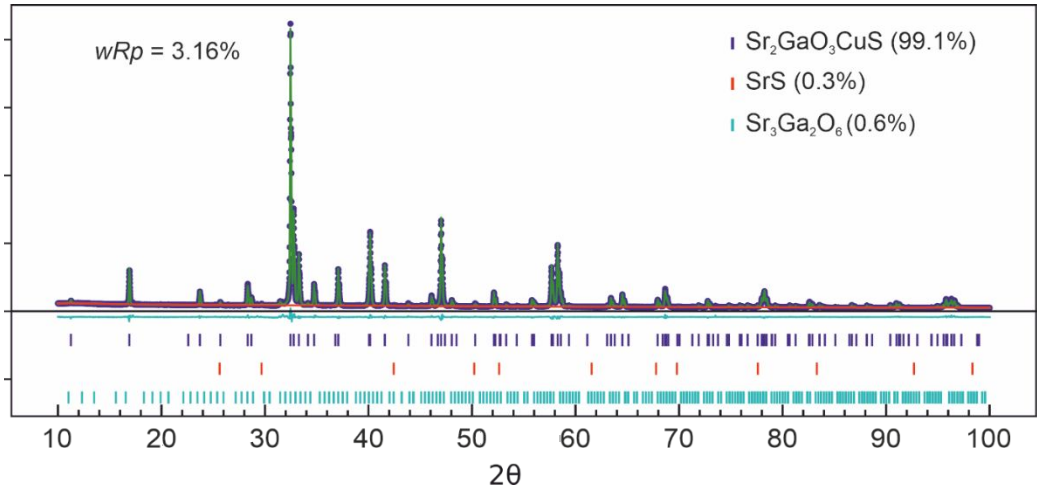

Figure S1. Rietveld refinement of Sr_2GaO_3CuS, data in blue, refinement fit in green.

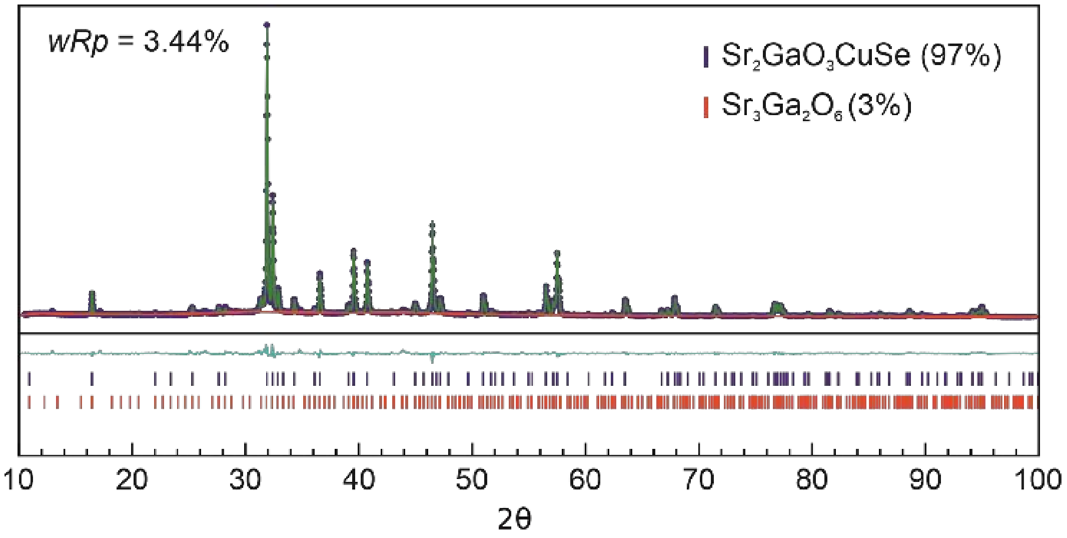

Figure S2. Rietveld refinement of Sr_2GaO_3CuSe, data in blue, refinement fit in green.

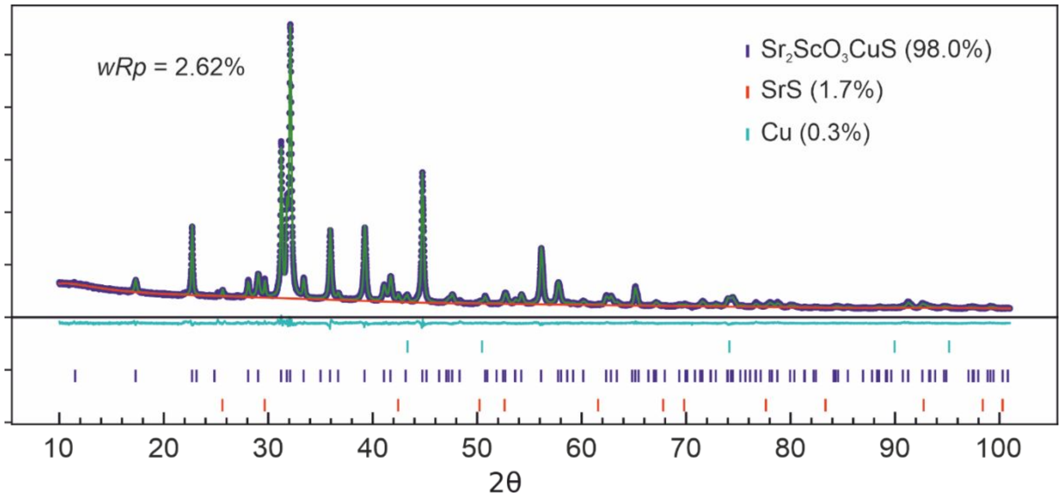

Figure S3. Rietveld refinement of Sr_2ScO_3CuS, data in blue, refinement fit in green.

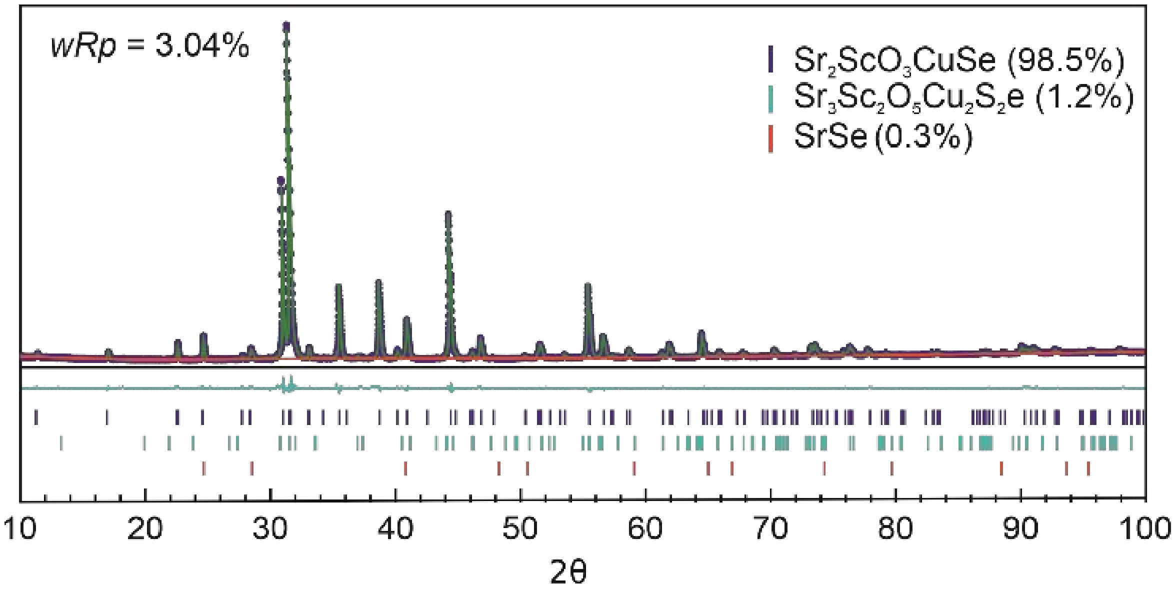

Figure S4. Rietveld refinement of Sr_2ScO_3CuSe, data in blue, refinement fit in green.

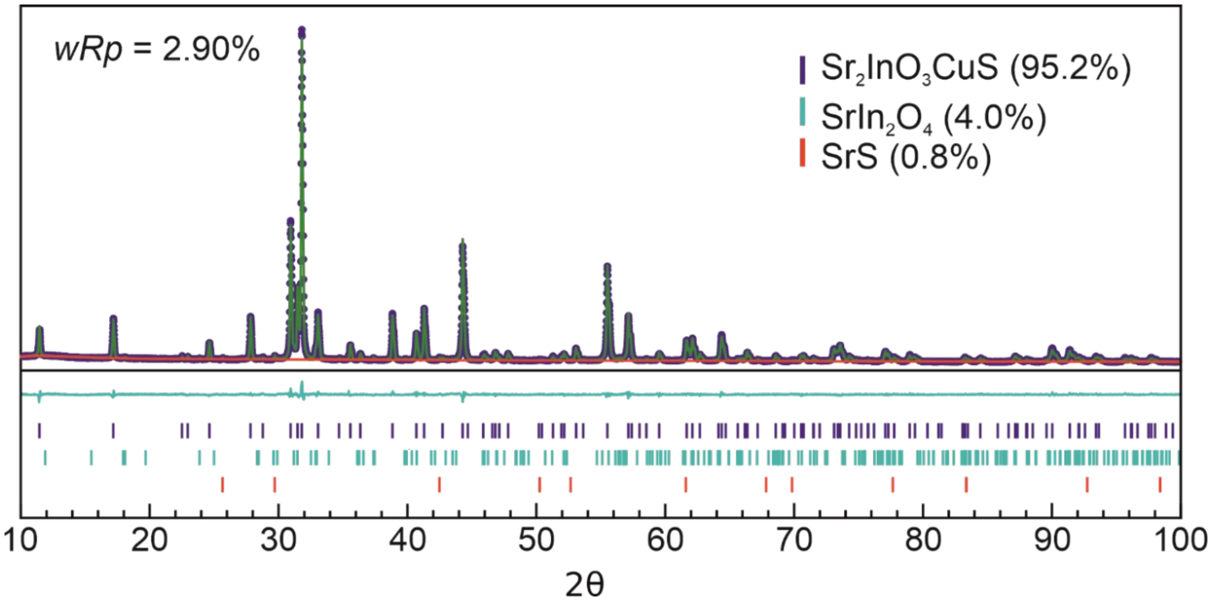

Figure S5. Rietveld refinement of Sr_2InO_3CuS, data in blue, refinement fit in green.

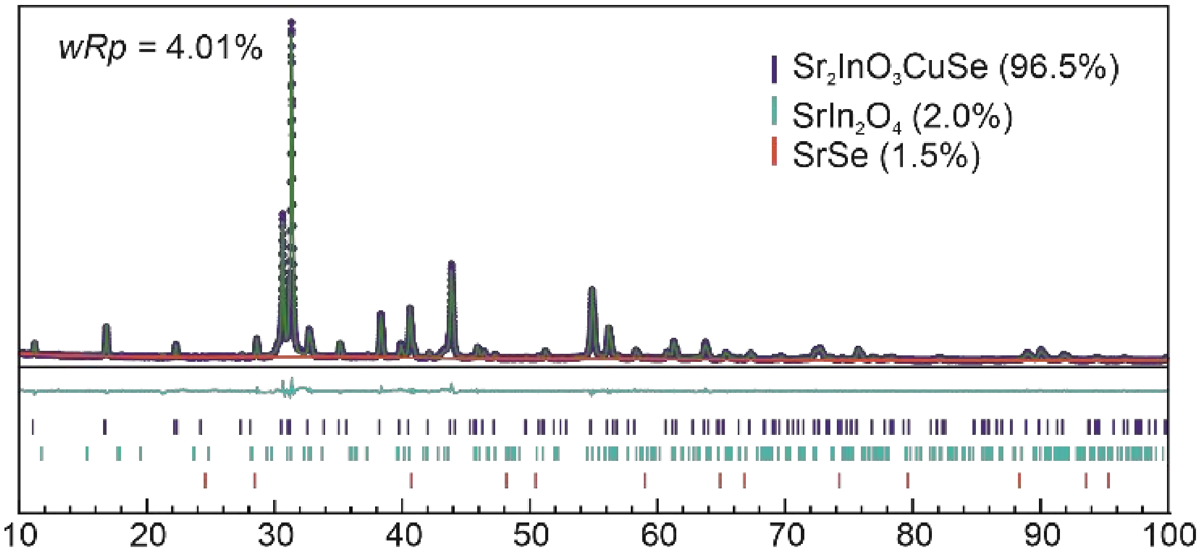

Figure S6. Rietveld refinement of Sr_2InO_3CuSe, data in blue, refinement fit in green.

Section B: Tables comparing our refined structural models to prior literature, where available

	$Sr_2Ga_2O_3CuS$, This work	$Sr_2Ga_2O_3CuS$, from reference 1
Lattice parameter a / Å	3.864026(13)	3.8606(4)
Lattice parameter c / Å	15.74911(6)	15.730(2)
Data Points	8887	N/A
Reflections (main phase)	104	N/A
Parameters	46	N/A
Purity	99.1	N/A
wRp	3.156	6.14
R_f^2	1.818	3.12
GoF	2.28	1.11
Sr1 (0.75, 0.75, z)	0.18435(5)	0.1842(2)
Sr2 (0.75, 0.75, z)	0.41401(5)	0.4140(1)
Ga (0.25, 0.25, z)	0.31210(7)	0.3144(2)
O1 (0.25, 0.75, z)	0.28940(16)	0.2899(4)
O2 (0.25, 0.25, z)	0.42975(22)	0.4270(6)
Cu (0.25, 0.75, z)	0	0
S (0.25, 0.25, z)	0.09413(15)	0.0940(3)

Table S1. Comparison of our refined model of Sr_3GaO_3CuS to prior literature data.

	Sr_2ScO_3CuS, this work	Sr_2ScO_3CuS, from reference 2
Lattice parameter a / Å	4.04780(4)	4.045
Lattice parameter c / Å	15.37012(20)	15.359
Data Points	8985	N/A
Reflections (main phase)	111	N/A
Parameters	44	N/A
Purity	98%	N/A
wRp	2.617	7.54
Rf2	2.476	2.88
GoF	1.61	N/A
Sr1 (0.75, 0.75, z)	0.17947(5)	0.181
Sr2 (0.75, 0.75, z)	0.41286(5)	0.415
Sc (0.25, 0.25, z)	0.30250(14)	0.305
O1 (0.25, 0.75, z)	0.28218(22)	0.283
O2 (0.25, 0.25, z)	0.43048(27)	0.429
Cu (0.25, 0.75, z)	0	0
S (0.25, 0.25, z)	0.08830(13)	0.09

Table S2. Comparison of our refined model of Sr_3ScO_3CuS to prior literature data.

	Sr_2InO_3CuS, this work	Sr_2InO_3CuS, from reference 3
Lattice parameter *a* / Å	4.092392 (20)	4.092009(38)
Lattice parameter *c* / Å	15.52813 (9)	15.51559(19)
Data Points	8887	N/A
Reflections (main phase)	111	N/A
Parameters	43	N/A
Purity	95.2%	100%
wRp	2.81	6.28
R_f^2	1.86	3.39
GoF	2.09	1.85
Sr1 (0.75, 0.75, *z*)	0.17706(5)	0.17598(19
Sr2 (0.75, 0.75, *z*)	0.41351(5)	0.41390(17)
In (0.25, 0.25, *z*)	0.30135(5)	0.30163(13)
O1 (0.25, 0.75, *z*)	0.27842(18)	0.28362(74)
O2 (0.25, 0.25, *z*)	0.4401(3)	0.4451(12)
Cu (0.25, 0.75, *z*)	0	0
S (0.25, 0.25, *z*)	0.08644(11)	0.08496(47)

Table S3. Comparison of the refined model of Sr_3InO_2CuS to prior literature data.

	Sr_2InO_3CuSe, this work	Sr_2InO_3CuSe from reference 3 [3]
Lattice parameter *a* / Å	4.12638(4)	4.125381(91)
Lattice parameter *c* / Å	15.82112(18)	15.81973(46)
Data Points	8887	N/A
Reflections (main phase)	115	N/A
Parameters	51	N/A
Purity	96.5%	93.6%
wRp	4.01	5.53
R_f^2	4.15	3.72
GoF	3.07	1.48
Sr1 (0.75, 0.75, *z*)	0.18483(16)	0.18464(24)
Sr2 (0.75, 0.75, *z*)	0.41576(14)	0.41492(20)
In (0.25, 0.25, *z*)	0.30547(15)	.30504(22
O1 (0.25, 0.75, *z*)	0.2843(4)	0.28354(86)
O2 (0.25, 0.25, *z*)	0.4397(7)	0.4409(14)
Cu (0.25, 0.75, *z*)	0	0
Se (0.25, 0.25, *z*)	0.09303(17)	0.09114(30)

Table S4. Comparison of the refined model of Sr_3InO_2CuSe to prior literature data.

Section C. Full structural details of all six Sr_2MO_3CuCh compounds, derived from samples and diffraction data prepared for this work

Atom Site	x	y	z	Uiso (Å^2)
Sr1	0.75	0.75	0.18435(5)	0.02885(27)
Sr2	0.75	0.75	0.41401(5)	0.02931(26)
Ga	0.25	0.25	0.31210(7)	0.02741(29)
O1	0.25	0.75	0.28940(16)	0.0571(11)
O2	0.25	0.25	0.42975(22)	0.0366(13)
Cu	0.25	0.75	0	0.0387(4)
S	0.25	0.25	0.09413(15)	0.0296(6)

Table S5. Structural parameters of the refined model of Sr_2GaO_3CuS. Sample refined in the *P4/nmm* space group, with a =3.864023(13) Å , c =15.74911(6) Å, vol =235.145(2) Å^3 , GOF = 2.28, Wrp = 3.16% and R_f^2=1.82% for 46 variables at room temperature.

Atom Site	x	y	z	Uiso (Å^2)
Sr1	0.75	0.75	0.17947(5)	0.0563(5)
Sr2	0.75	0.75	0.41286(5)	0.0553(5)
Sc	0.25	0.25	0.30250(14)	0.0430(5)
O1	0.25	0.75	0.28218(22)	0.0919(14)
O2	0.25	0.25	0.43048(27)	0.0430(14)
Cu	0.25	0.75	0	0.0785(6)
S	0.25	0.25	0.08830(13)	0.0593(9)

Table S6. Structural parameters of the refined model of Sr_2ScO_3CuS. Sample refined in the *P4/nmm* space group, with a =4.04780(4) Å c =15.37012(20) Å, vol =251.835(6) Å^3, GOF = 1.61, Wrp = 2.62% and R_f^2=2.48% for 46 variables at room temperature.

Atom Site	x	y	z	Uiso (Å^2)
Sr1	0.75	0.75	0.17706(5)	0.0110(4)
Sr2	0.75	0.75	0.41351(5)	0.0183(4)
In	0.25	0.25	0.30135(5)	0.00804(26)
O1	0.25	0.75	0.27842(18)	0.0272(12)
O2	0.25	0.25	0.4401(3)	0.0467(15)
Cu	0.25	0.75	0	0.0234(4)
S	0.25	0.25	0.08644(11)	0.0094(8)

Table S7. Structural parameters of the refined model of Sr_2InO_3CuS. Sample refined in the *P4/nmm* space group, with a = 4.092392 (20) Å, c = 15.52813 (9) Å, vol = 260.060(3) Å^3 , GOF = 2.09, Wrp = 2.811% and R_f^2=1.857% for 43 variables at room temperature.

Atom Site	x	y	z	Uiso (Å^2)
Sr1	0.75	0.75	0.19312(13)	0.0284(7)
Sr2	0.75	0.75	0.41598(11)	0.0323(9)
In	0.25	0.25	0.31667(14)	0.0275(10)
O1	0.25	0.75	0.2923(4)	0.0367(25)
O2	0.25	0.25	0.4334(6)	0.052(4)
Cu	0.25	0.75	0	0.0395(10)
Se	0.25	0.25	0.09785(12)	0.0268(9)

Table S8. Structural parameters of the refined model of Sr_2GaO_3CuSe. Sample refined in the *P*4/*nmm* space group, with a = 3.89988(4) Å, c = 16.09379(17) Å, vol = 244.771(6) Å^3, GOF = 3.72, Wrp = 3.30% and R_f^2=2.704% for 48 variables at room temperature.

Atom Site	x	y	z	Uiso (Å^2)
Sr1	0.75	0.75	0.18840(9)	0.0449(4)
Sr2	0.75	0.75	0.41500(7)	0.0431(4)
Sc	0.25	0.25	0.30730(18)	0.0344(6)
O1	0.25	0.75	0.28796(23)	0.0731(16)
O2	0.25	0.25	0.43136(28)	0.0636(19)
Cu	0.25	0.75	0	0.0567(5)
Se	0.25	0.25	0.09413(9)	0.0465(5)

Table S9. Structural parameters of the refined model of Sr_2ScO_3CuSe. Sample refined in the *P*4/*nmm* space group, with a = 4.076704(24) Å, c = 15.70839(13) Å, vol = 261.065(4) Å^3, GOF =2.25, Wrp = 3.23% and R_f^2=3.017% for 37 variables at room temperature.

Atom Site	x	y	z	Uiso (Å^2)
Sr1	0.75	0.75	0.18483(16)	0.0224(8)
Sr2	0.75	0.75	0.41576(14)	0.0396(10)
In	0.25	0.25	0.30547(15)	0.0247(6)
O1	0.25	0.75	0.2843(4)	0.0619(31)
O2	0.25	0.25	0.4397(7)	0.066(4)
Cu	0.25	0.75	0	0.0363(9)
Se	0.25	0.25	0.09303(17)	0.0211(9)

Table S10. Structural parameters of the refined model of Sr_2InO_3CuSe. Sample refined in the *P*4/*nmm* space group, with a = 4.12638(4) Å, c = 15.82112(18) Å, vol = 269.386(6) Å^3, GOF = 3.07, Wrp = 4.01% and R_f^2=4.153% for 51 variables at room temperature.

Bond / angle	Sr_2GaO_3CuS	Sr_2ScO_3CuS	Sr_2InO_3CuS	Sr_2GaO_3CuSe	Sr_2ScO_3CuSe	Sr_2InO_3CuSe
M-O1 eq / Å	1.96481(50)	2.04786(61)	2.07694(50)	1.9890(13)	2.06087(68)	2.0902(11)
M-O2 ax/ Å	1.8529(36)	1.9671(47)	2.1545(47)	1.8786(99)	1.9488(52)	2.124(11)
M-*X*/ Å	3.4328(26)	3.2923(29)	3.3372(19)	3.5216(30)	3.3486(32)	3.3610(36)
O1-*B*-O2 / °	100.484(87)	98.77(12)	99.871(87)	101.37(22)	98.48(13)	99.22(20)
*M*O5 block/ Å	2.2104(43)	2.2794(54)	2.5106(54)	2.271(12)	2.2526(57)	2.459(13)
Sr1-O1/ Å	2.5436(17)	2.5668(21)	2.5815(18)	2.5199(43)	2.5692(24)	2.5949(41)
Sr1-X / Å	3.0797(11)	3.18684(94)	3.21775(82)	3.1552(14)	3.24077(91)	3.2593(16)
O1_*A*1_X / °	79.839(42)	76.684(44)	76.266(37)	80.188(85)	77.240(48)	76.523(83)
Sr1 Block / Å	3.0753(35)	2.9800(39)	2.9811(33)	3.1294(67)	3.0448(39)	3.0261(69)
Sr2-O2 eq / Å	2.74350(32)	2.87501(40)	2.92307(67)	2.77185(99)	2.89410(40)	2.9423(15)
Sr2-O2-ax / Å	2.4606(36)	2.4079(42)	2.2732(47)	2.4240(98)	2.4134(45)	2.287(11)
Sr2 Block / Å	1.1064(35)	1.0685(42)	0.9301(47)	1.0718(97)	1.0782(44)	0.954(11)
Cu-*X* / Å	2.4352(14)	2.4368(11)	2.44715(94)	2.5064(12)	2.51818(83)	2.5344(16)
X-Cu-*X* / °	105.001(72)	112.310(61)	113.472(51)	102.151(58)	108.085(42)	108.993(79)
CuCh block / Å	2.9649(33)	2.7144(28)	2.6845(24)	3.1496(27)	2.9573(20)	2.9437(38)

Table S11. Summary of all major bond angles, bond lengths and 'block' heights for the Sr_2MO_3CuCh phases, where *M* = Sc, Ga or In, and *Ch* =S or Se. Graphical representation of the location of the different atom sites (*eg.* Sr1, Sr2) and 'blocks' please refer to figure 2 in the main paper.

Section D. Rietveld refinement fits and summary for sodium and potassium doped $A_{0.05}Sr_{1.95}MO_3CuCh$ samples

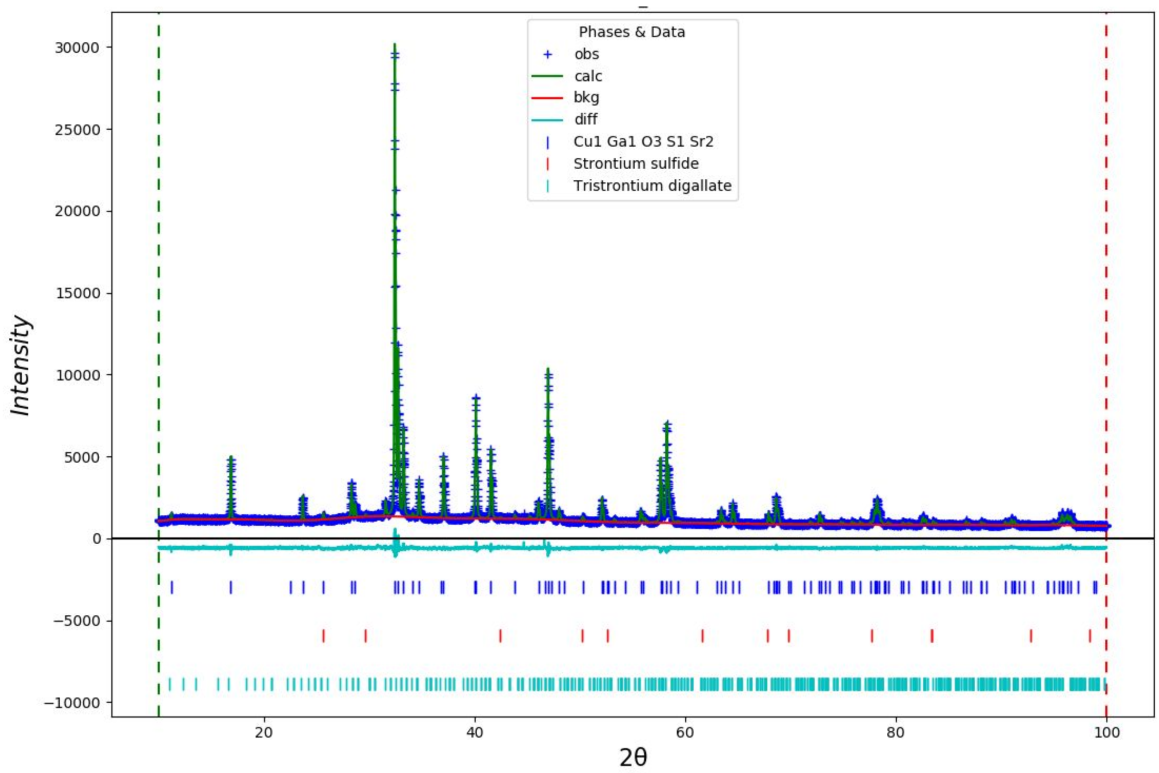

Figure S7. Refinement of sodium doped nominal $Na_{0.05}Sr_{195}GaO_3CuS$

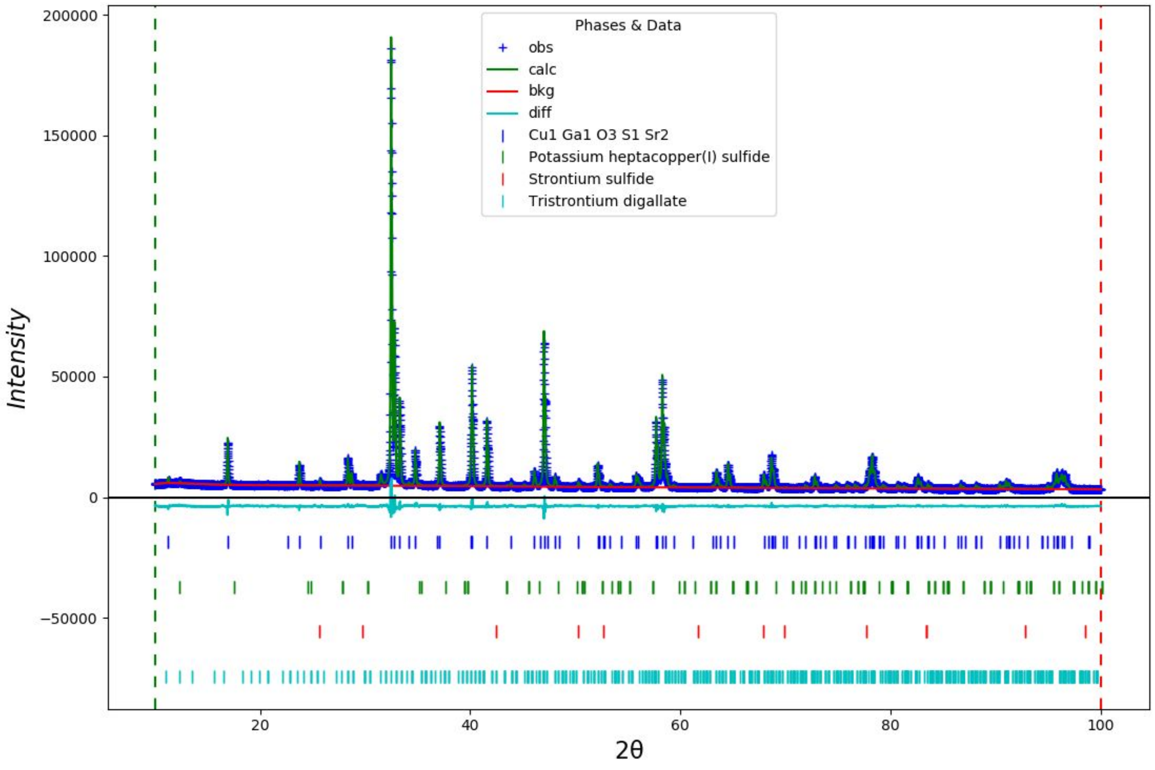

Figure S8. Refinement of potassium doped nominal $K_{0.05}Sr_{1.95}GaO_3CuS$

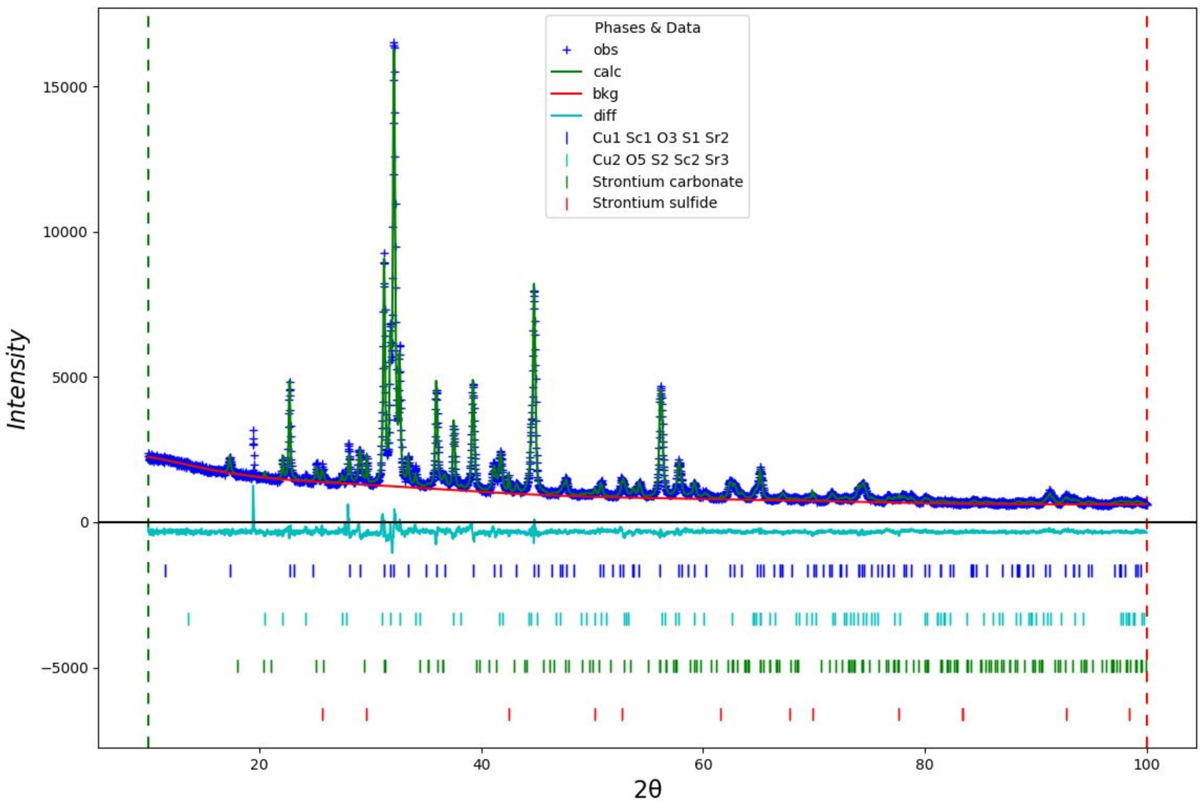

Figure S9. Refinement of sodium doped nominal $Na_{0.05}Sr_{1.95}ScO_3CuS$

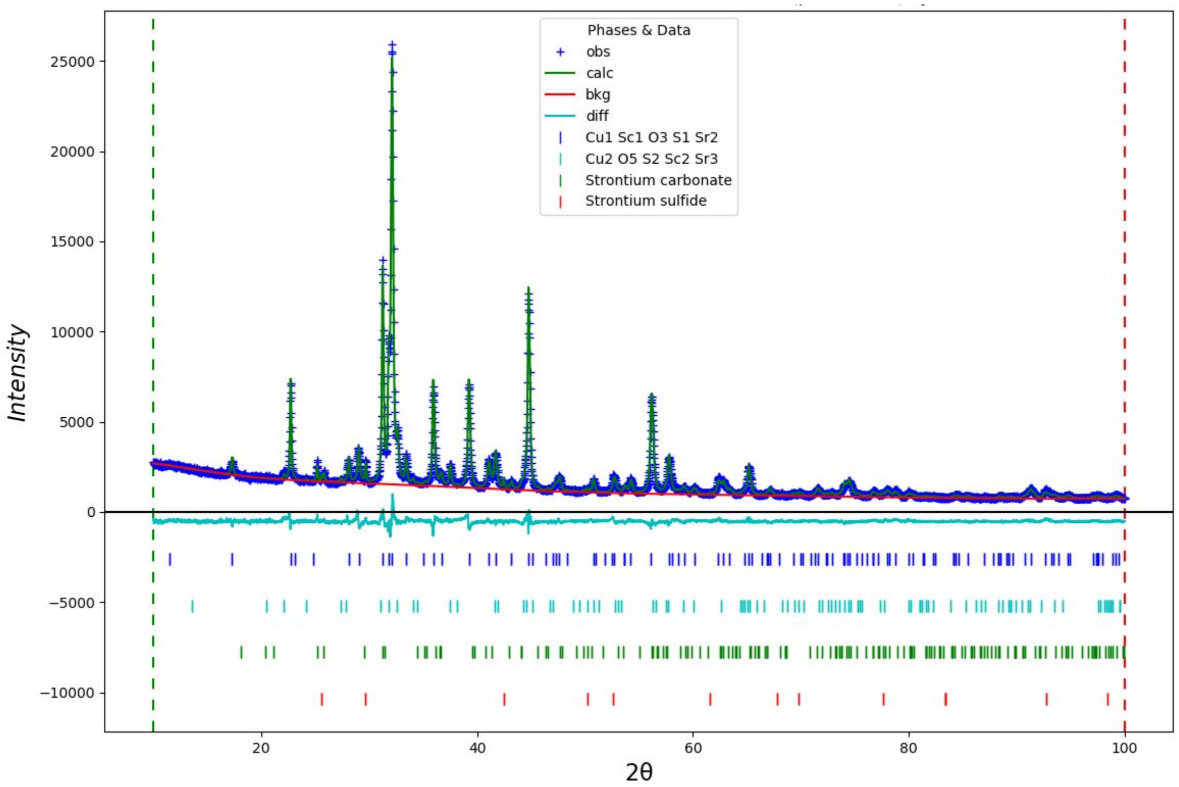

Figure S10. Refinement of potassium doped nominal $K_{0.05}Sr_{1.95}ScO_3CuS$

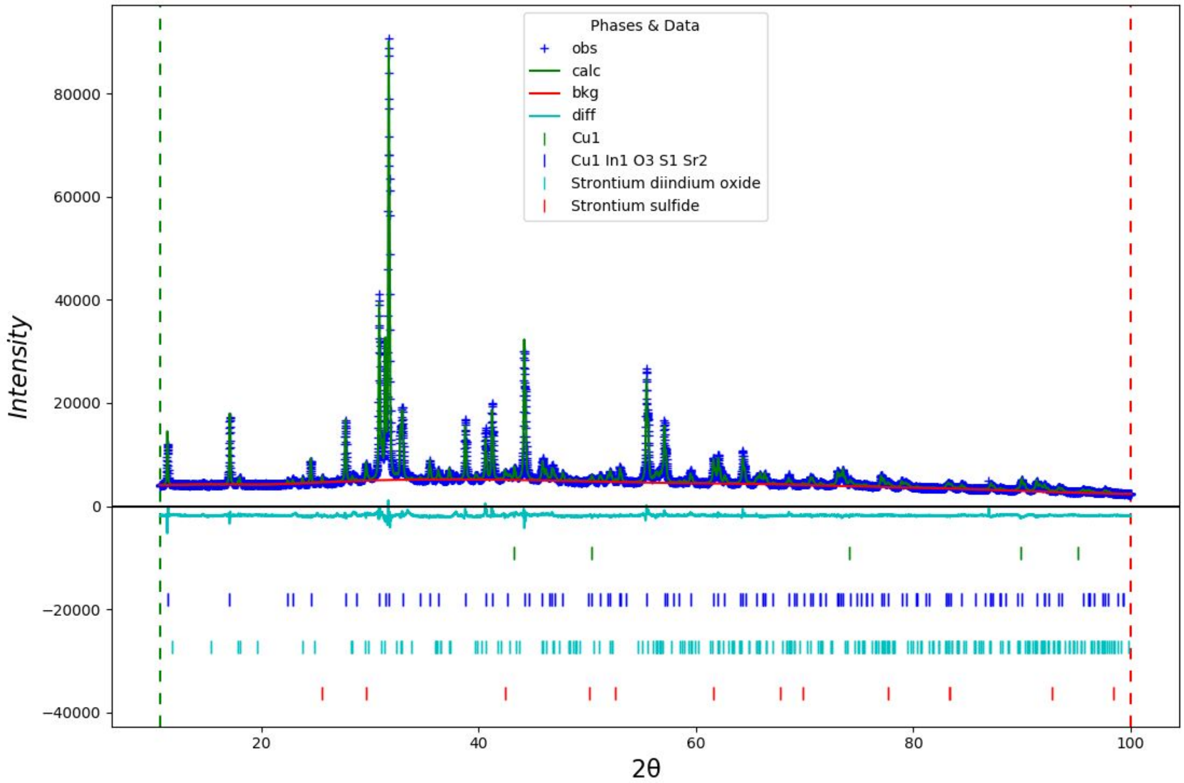

Figure S11. Refinement of sodium doped nominal $Na_{0.05}Sr_{1.95}InO_3CuS$

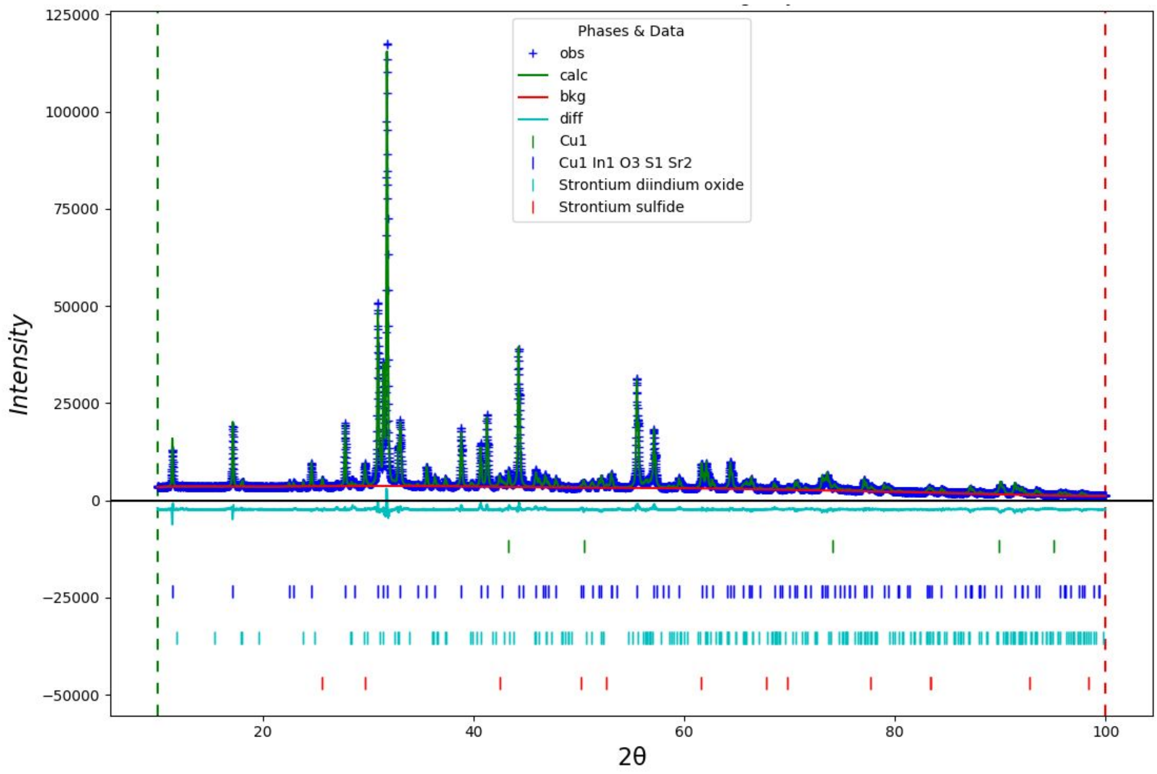

Figure S12. Refinement of potassium doped nominal $K_{0.05}Sr_{1.95}InO_3CuS$

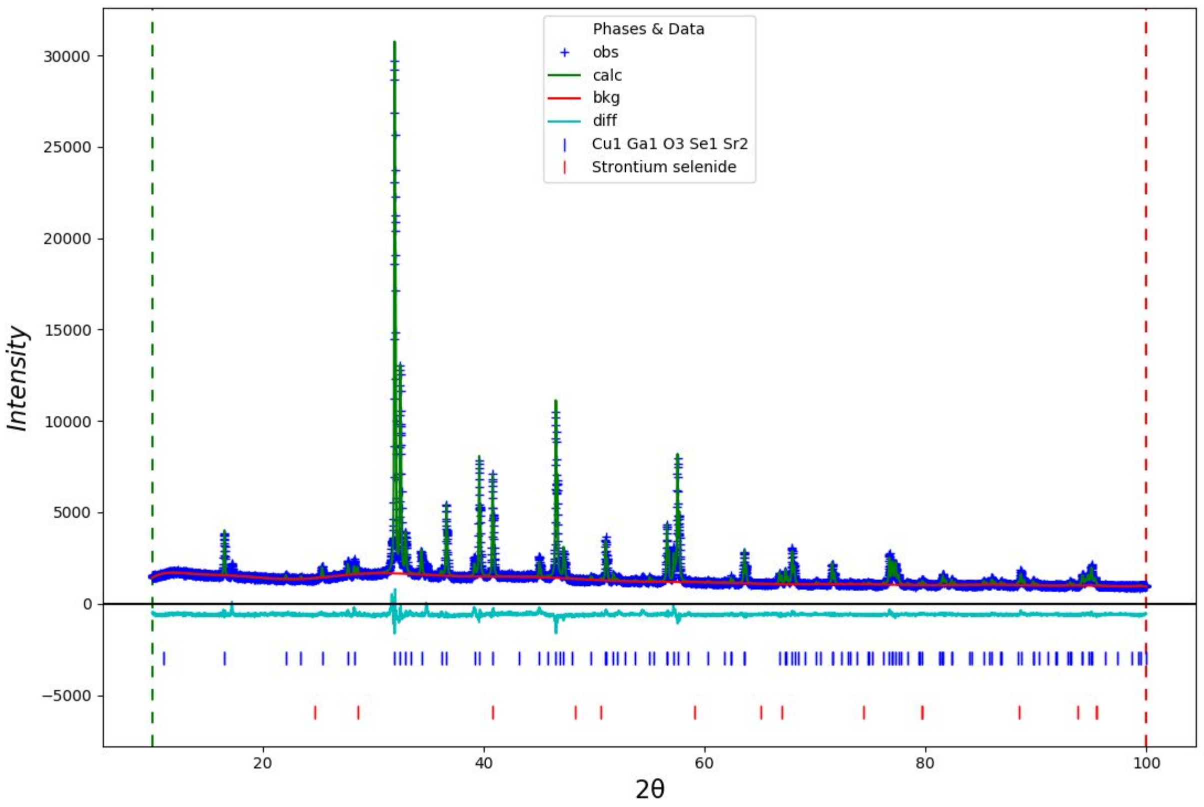

Figure S13. Refinement of sodium doped nominal $Na_{0.05}Sr_{1.95}GaO_3CuSe$

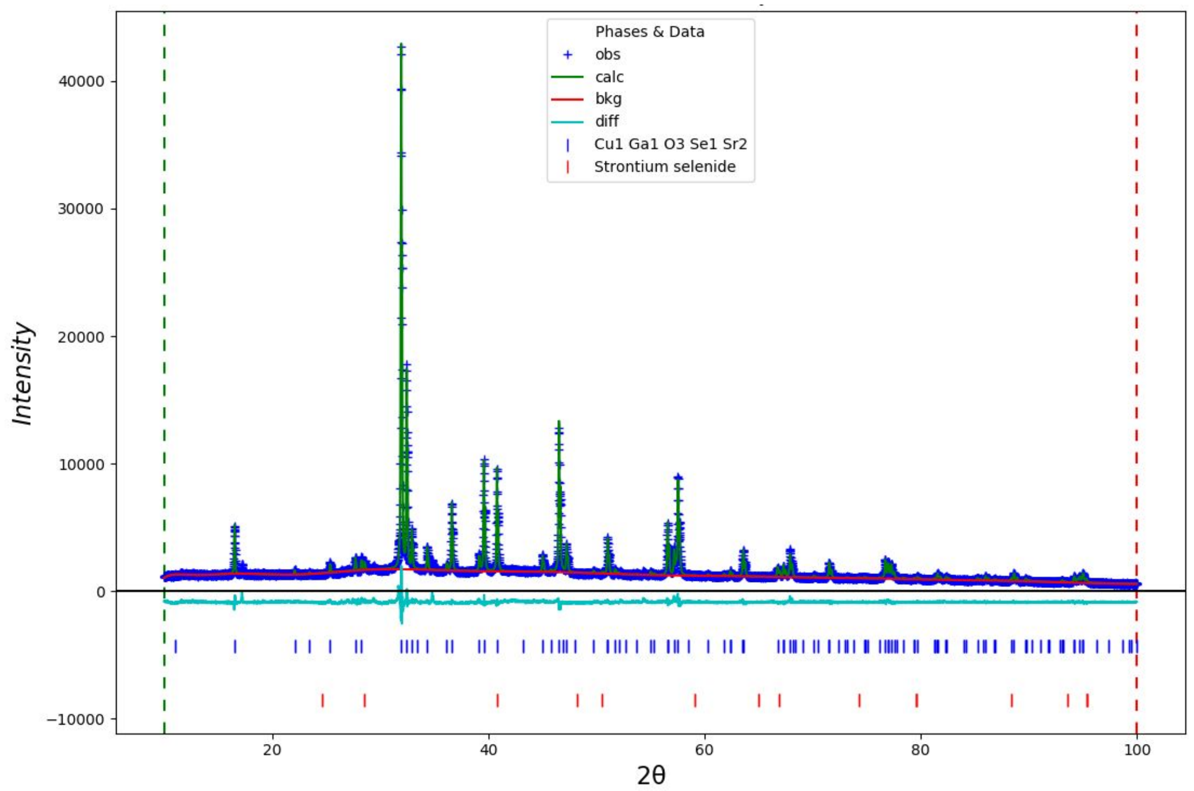

Figure S14. Refinement of potassium doped nominal $K_{0.05}Sr_{1.95}GaO_3CuSe$

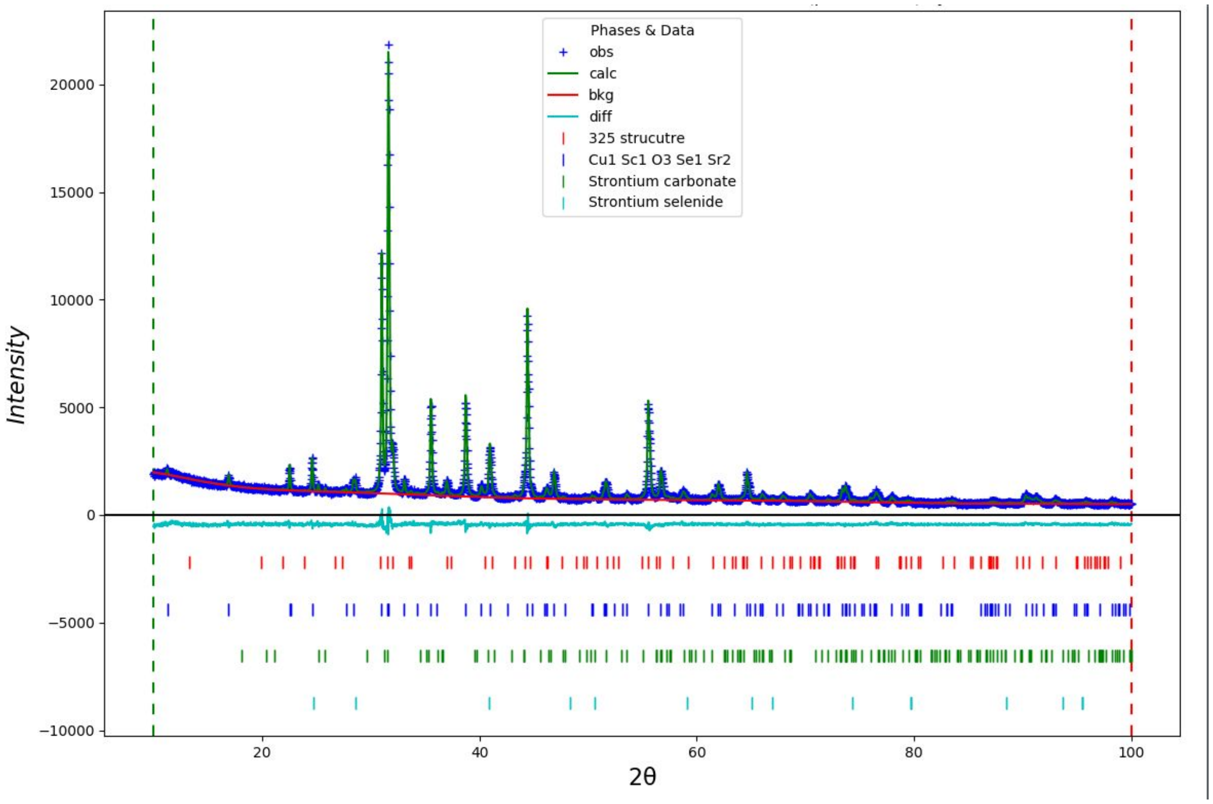

Figure S15. Refinement of sodium doped nominal $Na_{0.05}Sr_{1.95}ScO_3CuSe$.

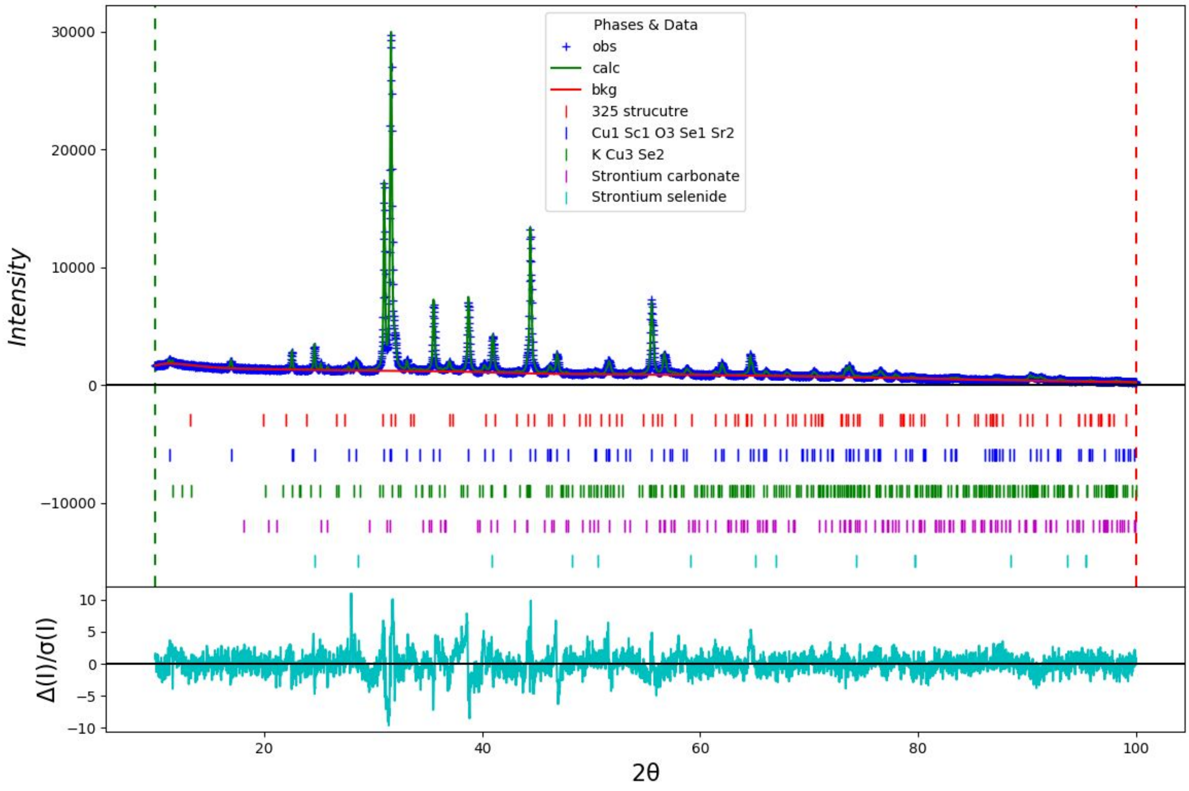

Figure S16. Refinement of potassium doped nominal $K_{0.05}Sr_{1.95}ScO_3CuSe$.

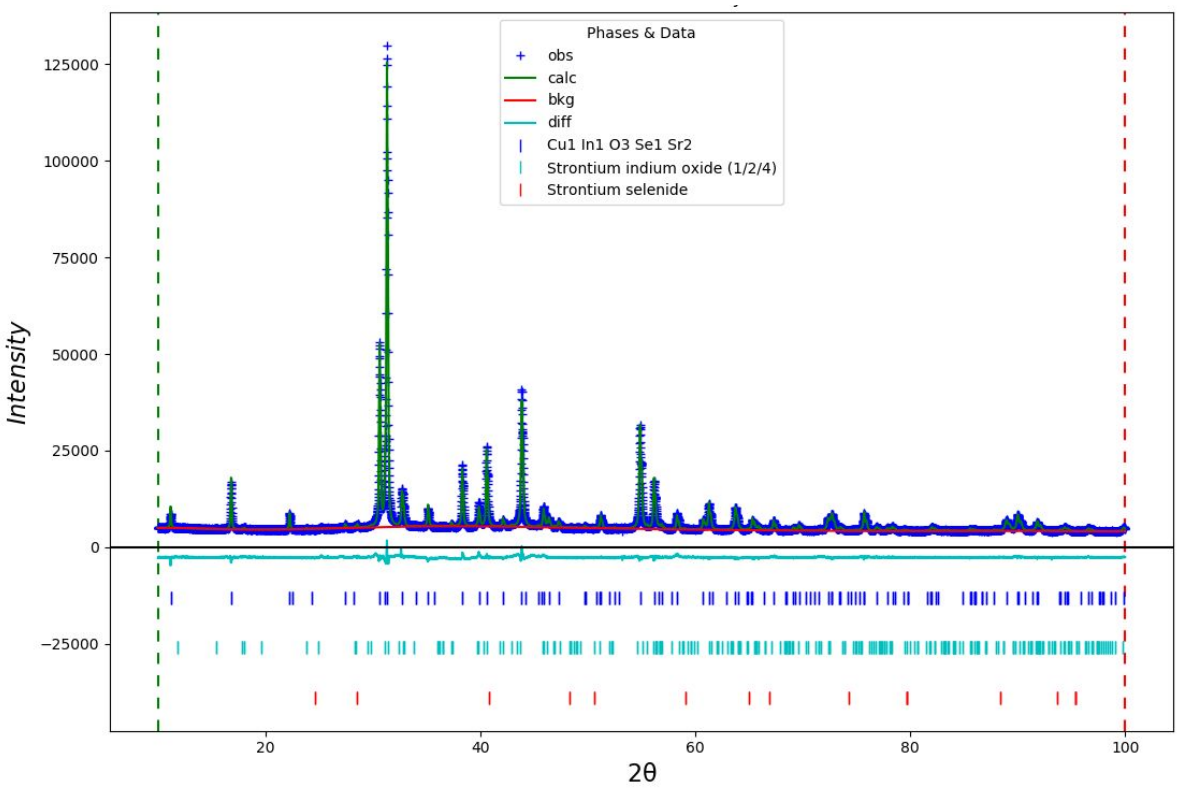

Figure S17. Refinement of sodium doped nominal $Na_{0.05}Sr_{1.95}InO_3CuSe$.

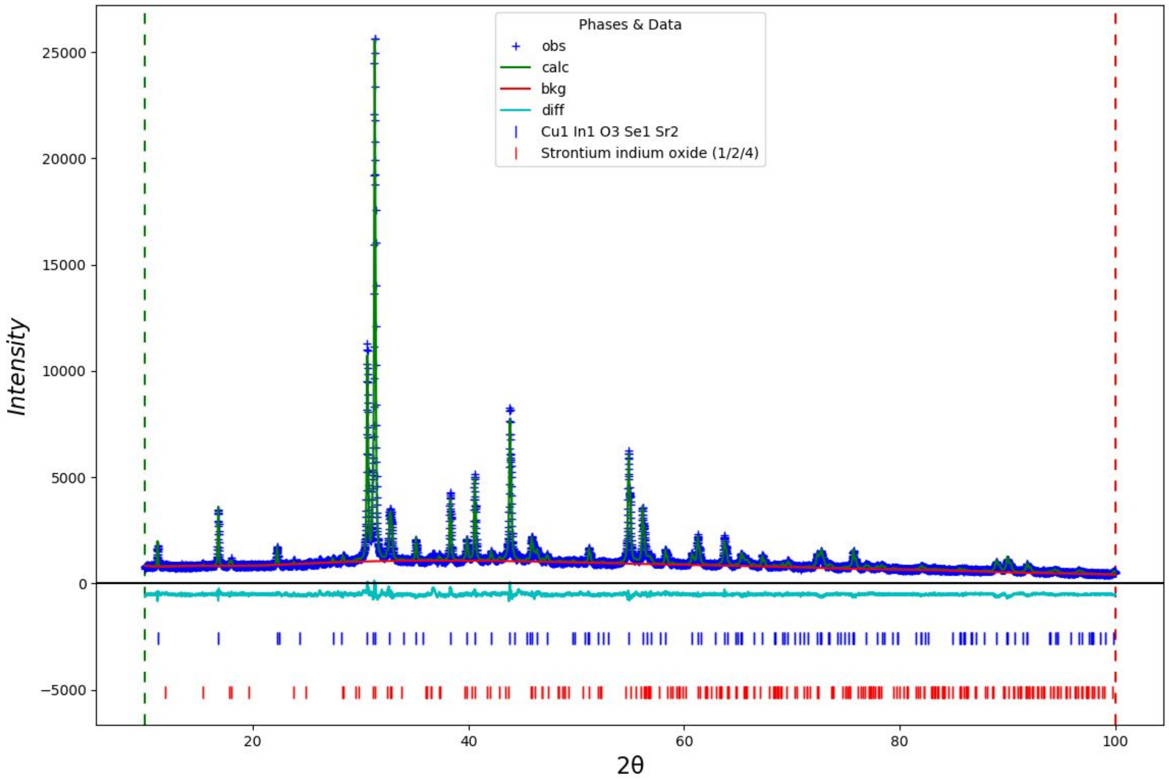

Figure S18. Refinement of potassium doped nominal $K_{0.05}Sr_{1.95}InO_3CuSe$.

Sample	Band Gap / eV	Results of refinement
Sr_2GaO_3CuS	2.43	wRp = 3.16%. 99.1 wt% main phase; 0.3 wt% SrS; 0.6wt% $Sr_3Ga_2O_6$
$Na_{0.05}Sr_{1.95}GaO_3CuS$	2.42	wRp = 3.62%. 98.1 wt% main phase; 0.3 wt% SrS ; 1.6 wt% $Sr_3Ga_2O_6$
$K_{0.05}Sr_{1.95}GaO_3CuS$	2.43	wRp 3.40%. 98.0 wt% main; 0.1 wt% SrS; 1. 7 wt% $Sr_3Ga_2O_6$; 0.7 wt% KCu_7S_4
Sr_2ScO_3CuS	3.15	wRp = 3.71%. 99.4 wt% main; 0.6 wt% $SrSO_4$
$Na_{0.05}Sr_{1.95}ScO_3CuS$	3.08	wRp = 5.01%. 83.1 wt% main; 0.8 wt% SrS; 13.7wt % $Sr_3Sc_2O_5Cu_2S_2$; 2.3wt % $SrCO_3$.
$K_{0.05}Sr_{1.95}ScO_3CuS$	3.11	wRp = 4.34%. 90.9 wt% main; 0.8 wt% SrS; 6.5 wt% $Sr_3Sc_2O_5Cu_2S_2$; 1.8w% $SrCO_3$
Sr_2InO_3CuS	2.29	wRp = 2.81%. 95.2 wt% main; 0.8 wt% SrS; 4 wt% $SrIn_2O_4$
$Na_{0.05}Sr_{1.95}InO_3CuS$	2.24	wRp = 3.57%. 83.1% main phase; 2.2 wt% SrS; 13.3 wt% $SrIn_2O_4$; **1.5 wt% Cu**
$K_{0.05}Sr_{1.95}InO_3CuS$	2.26	wRp = 4.59%. 84.8% main phase; 3.2% SrS; 10.1% $SrIn_2O_4$; **1.8% Cu**
Sr_2GaO_3CuSe	1.85	wRp = 3.3%. 97wt% main; 3 wt% $Sr_3Ga_2O_6$
$Na_{0.05}Sr_{1.95}GaO_3CuSe$	1.86	wRp = 4.6%. 99.1 wt% main; 0.4 wt% SrSe; 0.5 wt% $SrGa_2O_4$
$K_{0.05}Sr_{1.95}GaO_3CuSe$	1.85	wRp = 4.75%. 99.4 wt% main; 0.6 wt% SrSe
Sr_2ScO_3CuSe	2.95	wRp = 3.23%. 98.5 wt% main; 1.2 wt% $Sr_3Sc_2O_5Cu_2Se_2$; 0.3 w% SrSe.
$Na_{0.05}Sr_{1.95}ScO_3CuSe$	2.88	wRp = 4.1%. 90.3 wt% main; 8.2 wt% $Sr_3Sc_2O_5Cu_2Se_2$; 0.5 wt% SrSe; 1 wt% $SrCO_3$
$K_{0.05}Sr_{1.95}ScO_3CuSe$	2.94	wRp = 4.62%; 90.3 wt% main; 8.0 wt% $Sr_3Sc_2O_5Cu_2Se_2$; 0.3 wt% SrSe; 1.1 wt% $SrCO_3$; 0.3 wt% KCu_3Se_2
Sr_2InO_3CuSe	1.88	wRp = 4.01%. 96.5% wt% main; 1.5 wt% SrSe; 2 wt% $SrIn_2O_4$
$Na_{0.05}Sr_{1.95}InO_3CuSe$	1.87	wRp = 3.04%. 95.2 wt% main; 0.2 wt% SrSe; 4.6 wt% $SrIn_2O_4$
$K_{0.05}Sr_{1.95}InO_3CuSe$	1.84	wRp = 4.76%. 86.3 wt% main; 13.7 wt% $SrIn_2O_4$

Table S12. Summary of the band gaps and refinements on the doped layered oxychalcogenide phases. Data for undoped samples also provided.

References

(1) Zhu, W. J.; Hor, P. H. Sr2CuGaO3S, a Rare Example of Square Pyramidal Gallium. *Inorg. Chem.* **1997**, *36* (17), 3576-3577. DOI: 10.1021/ic970322c.

(2) Ogino, H.; Shimoyama, J.; Kishio, K.; Katsura, Y.; Tsuboi, M.; Yamanoi, K.; Cadatal-Raduban, M.; Nakazato, T.; Shimizu, T.; Sarukura, N. Excitonic luminescence in two-dimensionally confined layered sulfide oxides. *Appl. Phys. Lett.* **2012**, *101* (19), Article. DOI: 10.1063/1.4764941.

(3) He, X.; Cho, T.; Katase, T.; Hanzawa, K.; Kitani, S.; Hiramatsu, H.; Hosono, H.; Kamiya, T. Wide-Gap p-Type Layered Oxychalcogenides AE2CuInO3Ch (AE: Alkaline Earth; Ch: Chalcogen): Unusually Low Residual Carrier Concentration and Green-to-Red Emission. *Chem. Mater.* **2024**. DOI: 10.1021/acs.chemmater.4c00724.
